# Polycomb-Mediated Repression and Sonic Hedgehog Signaling Interact to Regulate Merkel Cell Specification during Skin Development

**DOI:** 10.1371/journal.pgen.1006151

**Published:** 2016-07-14

**Authors:** Carolina N. Perdigoto, Katherine L. Dauber, Carmit Bar, Pai-Chi Tsai, Victor J. Valdes, Idan Cohen, Francis J. Santoriello, Dejian Zhao, Deyou Zheng, Ya-Chieh Hsu, Elena Ezhkova

**Affiliations:** 1 Department of Developmental and Regenerative Biology, Black Family Stem Cell Institute, Icahn School of Medicine at Mount Sinai, New York, New York, United States of America; 2 Department of Stem Cell and Regenerative Biology, Harvard University and Harvard Stem Cell Institute, Cambridge, Massachusetts, United States of America; 3 Department of Neurology, Albert Einstein College of Medicine, Bronx, New York, United States of America; 4 Departments of Genetics and Neuroscience, Albert Einstein College of Medicine, Bronx, New York, United States of America; University of Bradford, UNITED KINGDOM

## Abstract

An increasing amount of evidence indicates that developmental programs are tightly regulated by the complex interplay between signaling pathways, as well as transcriptional and epigenetic processes. Here, we have uncovered coordination between transcriptional and morphogen cues to specify Merkel cells, poorly understood skin cells that mediate light touch sensations. In murine dorsal skin, Merkel cells are part of touch domes, which are skin structures consisting of specialized keratinocytes, Merkel cells, and afferent neurons, and are located exclusively around primary hair follicles. We show that the developing primary hair follicle functions as a niche required for Merkel cell specification. We find that intraepidermal Sonic hedgehog (Shh) signaling, initiated by the production of Shh ligand in the developing hair follicles, is required for Merkel cell specification. The importance of Shh for Merkel cell formation is further reinforced by the fact that Shh overexpression in embryonic epidermal progenitors leads to ectopic Merkel cells. Interestingly, Shh signaling is common to primary, secondary, and tertiary hair follicles, raising the possibility that there are restrictive mechanisms that regulate Merkel cell specification exclusively around primary hair follicles. Indeed, we find that loss of Polycomb repressive complex 2 (PRC2) in the epidermis results in the formation of ectopic Merkel cells that are associated with all hair types. We show that PRC2 loss expands the field of epidermal cells competent to differentiate into Merkel cells through the upregulation of key Merkel-differentiation genes, which are known PRC2 targets. Importantly, PRC2-mediated repression of the Merkel cell differentiation program requires inductive Shh signaling to form mature Merkel cells. Our study exemplifies how the interplay between epigenetic and morphogen cues regulates the complex patterning and formation of the mammalian skin structures.

## Author Summary

Merkel cells are innervated touch-receptor cells that are responsible for light touch sensations. They originate from embryonic epidermal stem cells and, in hairy regions of skin, are organized in touch domes. Touch domes are highly patterned structures that form exclusively around primary hair follicles. Strikingly, the mechanisms controlling Merkel cell formation are largely unknown. Here, we show that the hair follicle functions as a niche required for Merkel cell formation. We find that intraepidermal Sonic hedgehog (Shh) signaling, initiated by the production of Shh in the developing hair follicles, is required for Merkel cell specification, whereas Shh overexpression in embryonic epidermal progenitors leads to ectopic Merkel cells. Interestingly, Shh signaling is common to all hair types, suggesting that there are restrictive mechanisms that allow Merkel cell specification to occur exclusively around primary hairs. Indeed, we find that loss of Polycomb repressive complex 2 (PRC2) in the epidermis leads to the formation of ectopic Merkel cells around all hair types. We show that PRC2 loss expands the field of epidermal cells competent to differentiate into Merkel cells through derepression of key Merkel-differentiation genes; however, inductive Shh signaling is still required for the formation of mature Merkel cells. Our study illustrates how the interplay between epigenetic and morphogen cues functions to establish the complex patterning and formation of the mammalian skin.

## Introduction

The skin epithelium is an excellent model system to study mechanisms of stem cell maintenance and differentiation [1]. During skin development, a single layer of embryonic epidermal stem cells gives rise to multiple lineages, including the interfollicular epidermis (IFE), the hair follicles, and the Merkel cells [1,2]. The precise patterning of the skin suggests that there is crosstalk between different skin epithelial lineages. However, the precise mechanisms coordinating the development of skin structures are largely unknown.

Merkel cells are a subtype of mechanoreceptor cells involved in light touch sensations. Merkel cells are often organized in structures called touch domes. Touch domes are composed of Merkel cells and specialized keratinocytes, and are innervated by sensory neurons [2–8]. In humans, Merkel cell touch domes are localized in regions of high tactile acuity, either in glabrous skin or associated with hair follicles [2,9]. Similarly, in mice, Merkel cells are present in the glabrous epidermis of the paws, as well as in touch domes in the dorsal skin and in the outer root sheath of whisker hair follicles [2,9]. Much of our knowledge of the molecular mechanisms controlling Merkel cell development and homeostasis comes from the analysis of murine dorsal skin, where touch domes are organized in polarized, crescent-shaped, highly patterned structures that are located exclusively around primary hair follicles, which correspond to 1–3% of the mouse hair coat [2–8].

**Fig 1:**
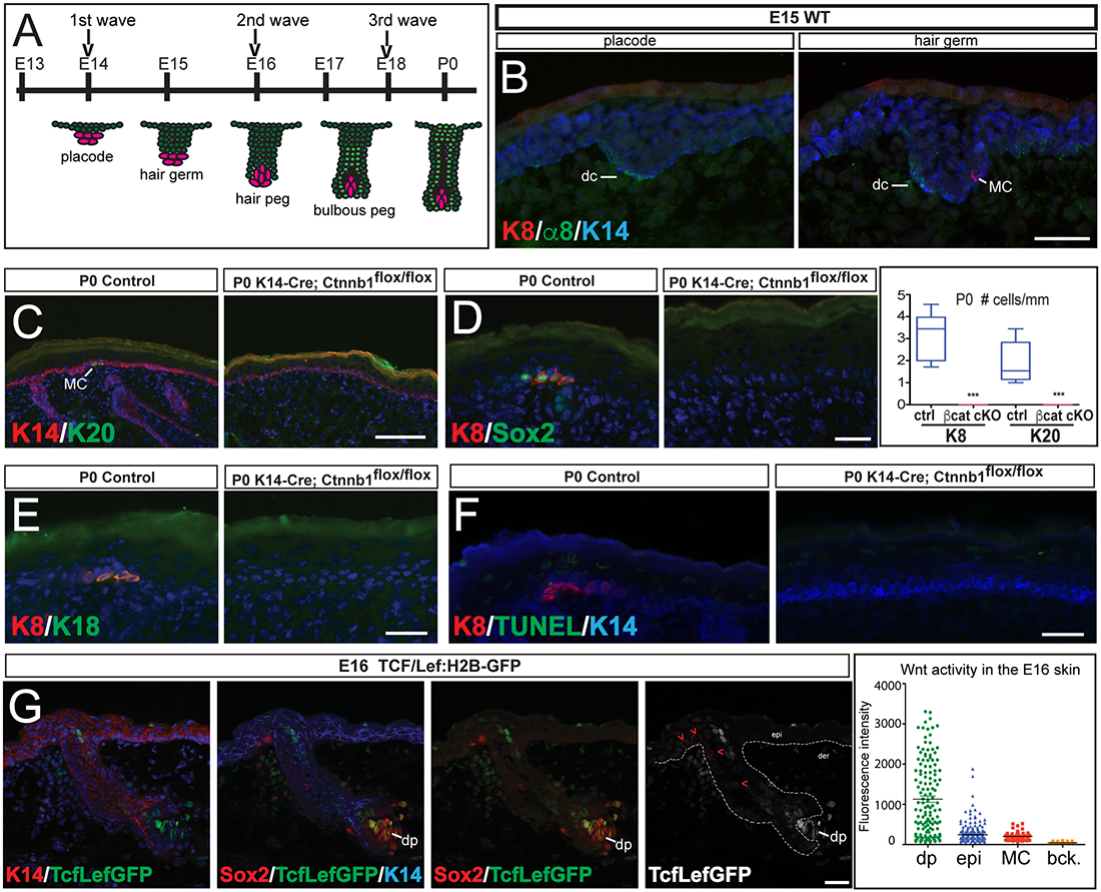
Loss of Wnt/β-catenin signaling abrogates hair follicle development and Merkel specification. (A) Schematic diagram of hair follicle development, with the different stages and the timing of each wave of hair follicle development represented. The epidermal cells are labeled in green and the mesenchymal cells that form the dermal condensate in the hair placode, germ, and peg stages, and the dermal papilla in the bulbous peg stage and the mature hair follicle are labeled in pink. (B) At E15, the first wave hair follicles are in the placode and hair germ stages. Early immature Krt8(+) (K8) Merkel cells (MC) are found around the WT first wave developing hair follicles at the hair germ stage (0% of placodes and 57.90% of hair germs contained Merkel cells; 100 hair follicles analyzed). Krt14 (K14) and integrin α8 (α8), which labels the dermal condensate (dc), are used to identify the developing hair follicles. (C-E) IF stainings for Merkel cell (MC) markers Krt20 (K20) (C), Krt8 (K8) (D,E), Sox2 (D), and Krt18 (K18) (E) show a complete absence of Merkel cells in P0 β-catenin epidermis-conditional knockout (β-cat cKO) (K14-Cre;Ctnnb1^flox/flox^) mice compared to control (ctrl). Quantification of Krt8+ and Krt20+ Merkel cells in control and β-cat cKO P0 skin (right panel of D) (both p<0.0001). (F) Terminal deoxynucleotidyl transferase dUTP nick end labeling (TUNEL) staining, which labels fragmented nuclear DNA, shows no increase in apoptosis in the Krt14(+) basal layer of P0 β-cat cKO mice. Note that cells undergoing cornification in the suprabasal layers stain positive for TUNEL as previously reported [33]. (G) The reporter of Wnt signaling activity, Tcf/Lef:H2B-GFP (TcfLefGFP), is used to measure Wnt signaling activity in the skin by measuring fluorescence intensity (right panel) in different cells of the skin. At E16, the Merkel cells do not have high levels of Wnt signaling. The dermal papilla (dp) cells are identified by Sox2 expression and are enveloped by the hair follicle; the epidermal cells are Krt14(+) (K14); the Merkel cells are Sox2(+) and are localized adjacent to the hair follicle. White dashed line separates the epidermis (epi) and dermis (der); red dashed line and arrowheads mark the Merkel cells. Non-nuclear background (bck) was measured. Scale bars: (C): 100μm; (B,D-G): 25 μm.

Previous studies have shown that the Wnt and Shh signaling pathways are essential for hair follicle development [10–21]. Mice in which Wnt signaling has been abrogated in the skin fail to develop hair follicles [15,16,22]. Wnt-dependent mesenchymal-epithelial signaling events induce hair follicle development, which is initiated by the formation of dense patches of epidermal cells, called hair placodes, and their associated dermal condensates (Fig 1A). Shh ligand is expressed in the hair placodes and Shh signaling is required for hair follicle down-growth, as in Shh KO mice, hair follicles remain arrested at the hair placode stage [11,20,21,23,24]. Other signaling pathways, such as Eda/Edar, FGF, and BMP signaling, also function to regulate hair follicle morphogenesis, acting downstream of Wnt or Shh signaling [25]. The importance of these signaling pathways for Merkel cell specification is largely unknown. There are three types of hair follicles, but only primary hairs contain Merkel cells. It is unknown, however, if the invariant localization of Merkel cells is mediated by primary hair follicle-associated signals controlling the setting up of the Merkel cell lineage and, if so, what those signals are. Furthermore, it is unknown whether the signaling mechanisms controlling Merkel cell formation in hairy skin also regulate Merkel cell development in the glabrous skin.

Once Merkel cells are specified, Merkel cell differentiation occurs through a maturation process characterized by the temporally regulated progressive expression of Merkel cell-specific genes, including those encoding transcription factors essential for Merkel differentiation (Atoh1, Sox2, and Isl1), cytoskeletal proteins (Krt8, Krt18, and Krt20), and components of the synaptic machinery [26]. Merkel cells and their associated neurons appear to develop independently, with Merkel cells only becoming innervated postnatally [27,28]. Furthermore, recent studies have shown that Polycomb repressive complex 2 (PRC2) restricts the Merkel cell differentiation program by repressing the *Sox2* and *Isl1* genes [29,30].

Here, we aimed to elucidate the molecular mechanisms regulating Merkel cell development. We show that hair follicle development is essential to Merkel cell formation, as abrogation of either Wnt or Shh signaling, which disrupts hair follicle formation, prevents Merkel cell specification. We find that Shh ligand produced by developing hair follicles is required to activate Shh signaling in the epidermis and for Merkel cell formation. Moreover, Shh overexpression in epidermal progenitors leads to ectopic Merkel cells, signifying the importance of Shh signaling for Merkel cell development. Interestingly, we show that in mice lacking PRC2 in the skin epidermis, Merkel cell specification is no longer restricted to primary hair follicles, but is now associated with all hair follicle types, indicating that all hair follicle types have the potential to induce Merkel cells. PRC2 does not affect essential hair follicle signaling pathways but instead restricts Merkel cell differentiation by repressing the expression of critical Merkel cell differentiation transcription factors in epidermal progenitor cells. Importantly, PRC2-null ectopic Merkel cells fail to form in the absence of Shh signaling. Finally, we show that inductive Shh signaling and repressive PRC2 mechanisms also control Merkel cell formation in the glabrous paw skin, indicating the global role of these signaling pathways in Merkel cell specification. Together, our data show that Polycomb-mediated repression imposes a restriction on the inductive Shh signaling that is essential for Merkel cell formation. Our study demonstrates how epigenetic and cell signaling cues interact to specify cell fate in a mammalian developmental system.

## Results

### Abrogation of hair follicle development results in Merkel cell loss

In murine wild type (WT) dorsal skin, Merkel cells are located in touch domes and found exclusively around primary hairs [2,31,32]. The primary hairs are formed during the first wave of hair follicle development, starting at embryonic day (E) 14 (Fig 1A) [2,31,32]. Analysis of Merkel cell formation in the dorsal skin revealed that early, immature Merkel cells are first detected at E15 and are localized around follicles that are in the hair germ stage. Merkel cells are not detected in the IFE, nor are they found in hair follicles at the earlier hair placode stage (Fig 1B) [26].

The spatial and temporal relationship between hair follicle and Merkel cell development prompted us to hypothesize that the developing hair follicle provides a local microenvironment required for Merkel cell formation. To test this, we started by analyzing the presence of Merkel cells in mice where Wnt/β-catenin signaling was conditionally ablated in the epidermis, abrogating hair follicle development prior to the formation of the hair placodes (S1A Fig) [15,16,18,25,34]. To generate these mice, we crossed mice carrying the conditional null allele of β-catenin (Ctnnb1^flox^) with mice expressing Cre recombinase under the control of the Krt 14 promoter, which is active in embryonic epidermal stem cells starting at E12 (β-cat cKO) [29,35]. Immunofluorescence staining for the β-catenin protein confirmed that it was lost in the β-cat cKO epidermis (S1B Fig) in neonate (P0) skin. Importantly, analysis of multiple Merkel cell markers revealed a complete absence of these cells in P0 β-cat cKO skin, while they were present in the control skin (Figs 1C–1E and S1C). We tested whether there was an increase in apoptosis by staining for Activated Caspase 3 and by Terminal deoxynucleotidyl transferase dUTP nick end labeling (TUNEL) staining, which labels fragmented nuclear DNA, detecting the extensive DNA degradation that occurs in apoptotic cells. TUNEL also labels cells undergoing cornification in the suprabasal layers of the interfollicular epidermis [33]. Combining Activated Caspase 3 and TUNEL analyses, we observed no increase in apoptosis in the P0 β-cat cKO epidermis, compared to control epidermis (Figs 1F and S1E). We measured proliferation by quantifying the number of cells positive for phosphorylated Histone H3 (PH3) in the epidermis. No alteration in proliferation was observed in the P0 β-cat cKO epidermis, compared to control epidermis (S1D Fig).

Since abrogation of Wnt/β-catenin signaling abolishes Merkel cell formation, we analyzed whether Merkel cells have active Wnt signaling. To do this, we used TCF/Lef1:H2B-GFP reporter mice, in which H2B-GFP is expressed in cells with active Wnt signaling [36], and analyzed H2B-GFP levels in Merkel cells at E16, during the early stages of Merkel cell formation. Interestingly, we observed that the H2B-GFP fluorescence signal in Sox2(+) Merkel cells was low and close to the background level, indicating that Merkel cells have low, if any active Wnt signaling (Fig 1G). In contrast, and consistent with previous reports, dermal papilla cells and some Krt14(+) epidermal cells have strong levels of Wnt signaling activity (Fig 1G) [22,37]. These data indicate that while Wnt/β-catenin signaling is required for Merkel cell formation, it likely functions indirectly in the control of this process.

### Hair follicle-derived Shh is required for Merkel cell formation during embryogenesis

Loss of β-catenin in the epidermis abrogates a cascade of signaling pathways that are normally activated during hair growth [25]. We speculated that one of the downstream pathways functions directly to specify Merkel cells. Shh signaling is one of the downstream pathways that are activated by Wnt signaling in the epidermis [25]. We therefore investigated whether Shh signaling is required for Merkel cell specification. We started by analyzing the presence of Merkel cells in the skin of sonic hedgehog (Shh)-null mice (Shh KO). To generate these mice, we crossed mice carrying the Shh^EGFPcre^ allele, a null *Shh* allele [38]. As Shh KO mice have severe developmental defects, including an absence of fully developed limbs, and die soon after birth [21,39], we performed our analysis at embryonic time points. *In situ* hybridization analysis of *Shh* mRNA confirmed the loss of Shh expression in Shh KO mice (S2A Fig) and, as previously reported [20,21,25,34], in E18 Shh KO mice, hair follicle development is arrested at the placode stage (S2B Fig).

**Fig 2:**
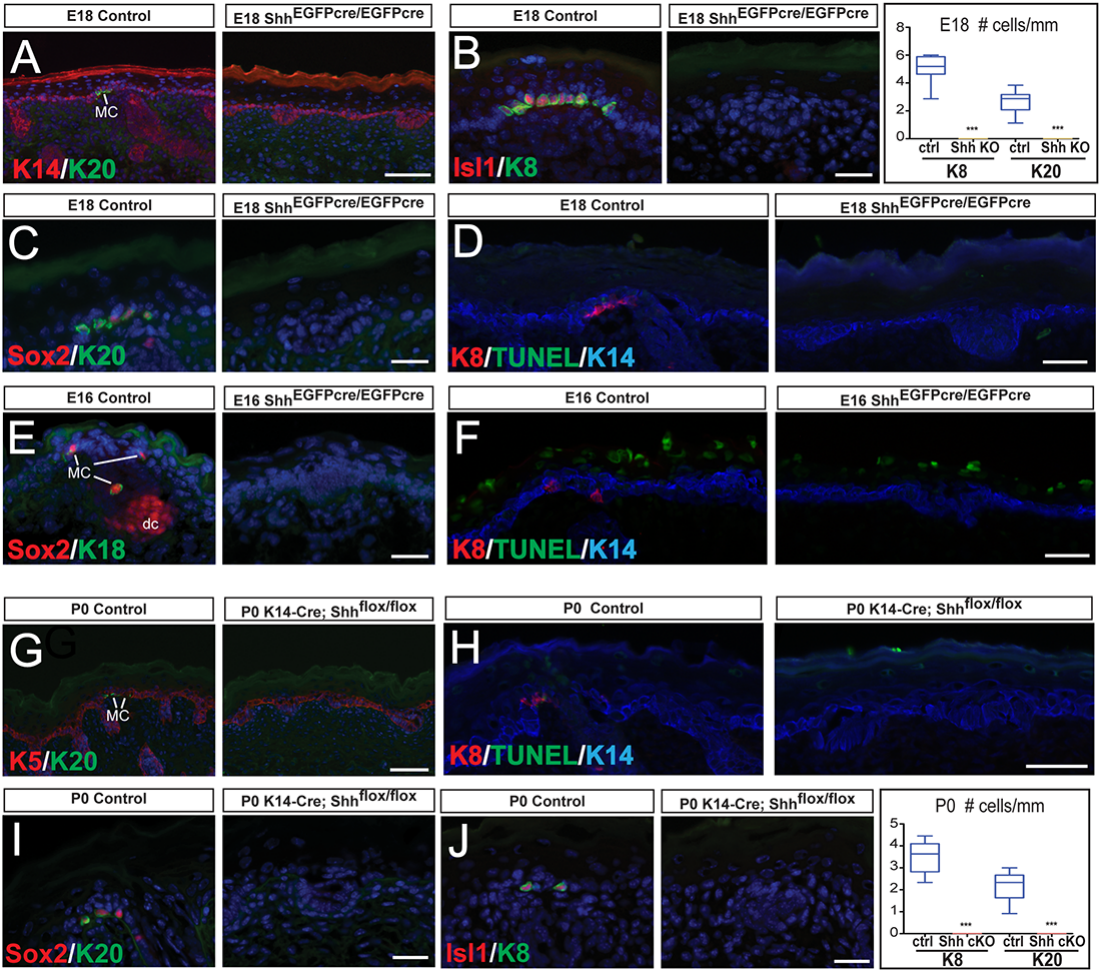
Shh signaling activity is required for Merkel cell formation. (A-C) IF stainings for Merkel cell (MC) markers Krt20 (K20) (A,C), Isl1 (B), Krt8 (K8) (B), and Sox2 (C) show a complete absence of Merkel cells in E18 Shh KO (Shh^EGFPcre/EGFPcre^) mice compared to control (ctrl). Quantification of Krt8(+) and Krt20(+) Merkel cells in control and Shh KO E18 skin (right panel of B) (Krt8 p = 0.0005 and Krt20 p = 0.0001). (D) TUNEL staining shows no increase in apoptosis in the skin of E18 Shh KO mice. (E) IF stainings for Merkel cell markers Krt18 (K18) and Sox2 show a complete absence of Merkel cells in E16 Shh KO mice when compared to control. Sox2 also labels the dermal condensate (dc). (F) TUNEL staining shows no increase in apoptosis in the Krt14(+) basal layer of E16 Shh KO mice when compared to control. Note that cells undergoing cornification in the suprabasal layers are TUNEL(+), as previously reported [33]. (G,I,J) IF stainings for Merkel cell (MC) markers Krt20 (G,I), Krt8 (J), Isl1 (J), and Sox2 (I) show a complete absence of Merkel cells in P0 Shh epidermis-conditional knockout (Shh cKO) (K14-Cre; Shh^flox/flox^) mice compared to control. Quantification of Krt8(+) and Krt20(+) Merkel cells in control and Shh cKO P0 skin (right panel of J) (both p<0.0001). (H) TUNEL staining shows no increase in apoptosis in the Krt14(+) layer P0 K14-Cre; Shh^flox/flox^ mice. Note that cells undergoing cornification in the suprabasal layers are TUNEL(+). Scale bars: (A,G): 100μm; (B-F; H-J): 25 μm.

Immunofluorescence analysis of Merkel cell markers revealed a complete absence of Merkel cells in E18 Shh KO skins (Fig 2A–2C). No increase in apoptosis or changes in cell proliferation were observed in Shh KO skins (Figs 2D and S2C and S2D). Analysis of an earlier time point, E16, also revealed loss of Merkel cells (Figs 2E and S2E) and no increase in the number of apoptotic cells (Figs 2F and S2F), indicating that Merkel cells are not specified in Shh KO embryos and are not dying by apoptosis.

In the skin, there are multiple sources of Shh expression, including the hair follicles, and the neurons innervating the skin [23,24,40]. Since our data show that Shh is required for Merkel cell formation, we analyzed whether the Shh ligand that is produced by the hair placodes is required for this process. To test this, we generated Shh epidermis-conditional knockout mice (Shh cKO) by crossing Shh^flox^ mice with Krt14-Cre mice. *In situ* hybridization of *Shh* mRNA confirmed loss of Shh expression in P0 Shh-null hair follicles compared to control epidermis (S2G Fig). Similar to those of Shh KO mice, Shh cKO hair follicles were arrested at the placode stage (Fig 2G), and there were no significant alterations in apoptosis or proliferation between P0 Shh cKO and control epidermis (Figs 2H, S2I and S2J). Importantly, by performing immunofluorescence analysis of multiple Merkel cell markers, we detected a complete absence of Merkel cells in P0 Shh cKO dorsal skin (Figs 2G, 2I, 2J and S2H), indicating that the Shh produced by developing hair follicles is required for Merkel cell formation during development.

### Shh signaling is required in the epidermis for Merkel cell specification

The Shh produced by the hair placodes activates Shh signaling both in the mesenchymal dermal papilla and in the epidermis and, in both of these compartments, active Shh signaling is required for proper hair follicle formation [11,20,21,25]. Since the Shh produced by the developing hair follicle is essential for Merkel cell development, we tested whether Shh signaling in the epidermis is required for the specification of the Merkel cells. To address this, we generated epidermis-conditional knockout mice of Smoothened (Smo cKO), an essential Shh-signaling signal transducer, by crossing Smo^flox^ mice with Krt14-Cre mice.

**Fig 3:**
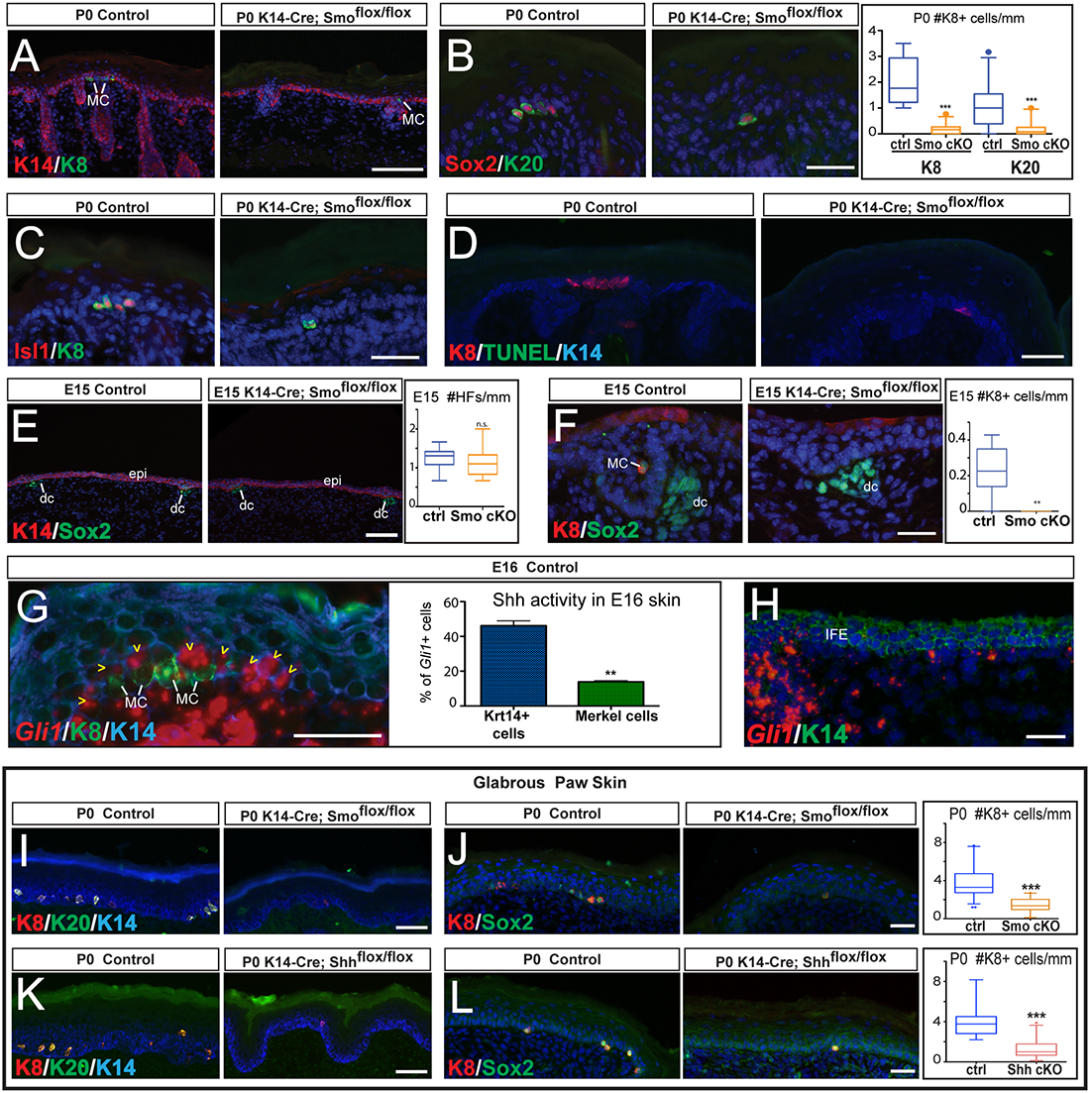
Shh signaling activity in the epidermis is required for Merkel cell formation. (A-C) IF stainings for Merkel cell markers Krt8 (K8) (A,C), Krt20 (K20) (B), Sox2 (B), and Isl1 (C) show a highly significant reduction in the number of Merkel cells in P0 Smoothened epidermis-conditional knockout (Smo cKO) (K14-Cre; Smo^flox/flox^) mice when compared to control (ctrl). Quantification of Krt8(+) and Krt20(+) Merkel cells in control and Smo cKO P0 skin (right panel of B) (both p<0.0001). (D) TUNEL staining shows no increase in apoptosis in the skin of P0 Smo cKO mice. Note that cells undergoing cornification are TUNEL(+) as previously reported [33]. (E) At E15, specification of hair follicles is not affected in P0 Smo cKO mice when compared with control. The number of hair follicles is quantified in the right panel (p = 0.3140). IF staining for Krt14 (K14) labels the epidermis (epi) and Sox2 labels the dermal condensate (dc) of the developing hair follicles in E15 WT and Smo cKO mice. (F) IF staining for Merkel cell markers Krt8 and Sox2 shows a complete absence of Merkel cells in E15 Smo cKO compared to control. The number of Krt8(+) Merkel cells is quantified in the right panel (p = 0.0018). (G) IF stainings for Merkel cell markers Krt8 and *in situ* hybridization for *Gli1* RNA in E16 WT skin indicates that *Gli1* is expressed in about 45% of the Krt14(+) (K14) cells (yellow arrowheads) surrounding the Merkel cells and in about 13% of early Merkel cells present in the skin at this time point, quantified in the right panel (p = 0.0022). (H) *In situ* hybridization for *Gli1* RNA in E16 WT back skin indicates that *Gli1* is not expressed in most Krt14(+) cells of the interfollicular epidermis (IFE). (I,J) IF stainings for Merkel cell markers Krt8 (I,J), Krt20 (I), and Sox2 (J) show that significantly fewer Merkel cells are present in the glabrous paw skin of Smo cKO mice compared to control paws. Quantification of Krt8(+) Merkel cells in paws (right panel of J) (p<0.0001). (K,L) IF stainings for Merkel cell markers Krt8 (K,L), Krt20 (K), and Sox2 (L) show that significantly fewer Merkel cells are present in the glabrous paw skin of Shh cKO mice compared to control paws. Quantification of Krt8(+) Merkel cells in paws (right panel of L) (p<0.0001). Unless otherwise indicated, all epidermis represented is dorsal skin. Scale bars: (A,E): 100μm; (B-D,F-L): 25 μm.

Consistent with previous reports, Smo-null hair follicles failed to develop properly, presenting abnormal growth and morphogenesis (Figs 3A and S3A) [41]. Importantly, immunofluorescence analysis of Merkel cell markers revealed a drastic reduction in the number of Merkel cells in neonatal Smo cKO skin compared to control skin (Fig 3A–3C), although a few fully differentiated Merkel cells were observed in P0 Smo cKO skin (Fig 3A–3C). Analysis of cell proliferation and apoptosis did not reveal significant alterations between control and Smo cKO skins at P0 (Figs 3D, S3B and S3C). Analysis of Smo cKO epidermis 9 days after birth (P9) revealed that hair follicle morphogenesis is grossly affected (S3D Fig) [41]. While there was no increase in apoptosis in these mice (S3E Fig), Merkel cells were nearly completely absent from the P9 Smo cKO dorsal skin (S3F Fig). This suggests that the observed phenotype in Smo cKO skin was not due to delay of Merkel cell formation.

While hair follicle development is severely affected in Smo-null epidermis, the initial steps of hair follicle specification at E15 are not affected in the Smo cKO (Fig 3E) [41]. Importantly, no Merkel cells could be detected in the Smo cKO epidermis at this time point, when hair follicles are normally specified (Fig 3E and 3F). Analysis of apoptosis did not reveal significant alterations between control and Smo cKO skins at E15 (S3G and S3H Fig). Thus, we concluded that intraepidermal Shh signaling is required for Merkel cell formation.

Gli1 is an effector of Shh signaling that can be used to detect Shh signaling activity in cells [11,42]. By analyzing β-galactosidase staining in the developing skin of Gli1-LacZ reporter mice [42] and *Gli1 in situ* hybridization in WT skins, we observed that Gli1 expression is strong in the developing hair follicles and is also detected in the epidermis near hair follicles, as well as in the dermis (S3I–S3J’ Fig). Next, we analyzed whether Merkel cells have active Shh signaling activity by performing *Gli1 in situ* hybridization, followed by immunofluorescence staining for an early Merkel cell marker, Krt8. We found that 14% of Merkel cells were *Gli1(*+) (Fig 3G), suggesting that while the Shh pathway might be active in some Merkel cells, Shh signaling activity is not detectable in most Merkel cells. As lineage tracing analysis has shown that Merkel cells arise from Krt14(+) epidermal stem cells during development [43,44], we analyzed *Gli1* expression and found that 46% of the Krt14(+) cells that were in the vicinity of the Merkel cells express *Gli1* (Fig 3G), while *Gli1* was rarely detected in the Krt14(+) cells located in the epidermis between the hair follicles (Fig 3H).

### Intraepidermal Shh signaling is required in the glabrous epidermis for Merkel cell specification

Merkel cells are localized not only in hairy skin but also in glabrous skin, such as paw epidermis. To test whether the intraepidermal Shh signaling is globally required for Merkel cell development, we analyzed the presence of Merkel cells in the glabrous paw epidermis of Smo cKO mice. In order to quantify the number of Merkel cells in the paws, we measured the length of the Krt14(+) epidermis and counted the number of Merkel cells localized in the different regions of the paws (S4A Fig). The analysis of paws of P0 Smo cKO mice revealed a very significant reduction in the number of Merkel cells compared to control (Fig 3I and 3J). No increase in apoptosis was detected in the glabrous paw epidermis of Smo cKO mice (S4B Fig). The fact that the number of Merkel cells was reduced to about half in the Smo cKO paws suggests that intraepidermal Shh signaling is important for Merkel specification in the glabrous skin, but that other signaling pathways are also likely to play a role in Merkel cell formation in the glabrous skin.

Finally, we investigated whether epidermis-derived Shh is important for Merkel cell formation in the paw epidermis, as we observed in the hairy skin. We thus analyzed paws of P0 Shh cKO mice and found that while Merkel cells where still present, there was a very significant reduction in the number of Merkel cells in Shh cKO, compared to control paws (Fig 3K and 3L).

### Shh signaling promotes Merkel cell specification

**Fig 4:**
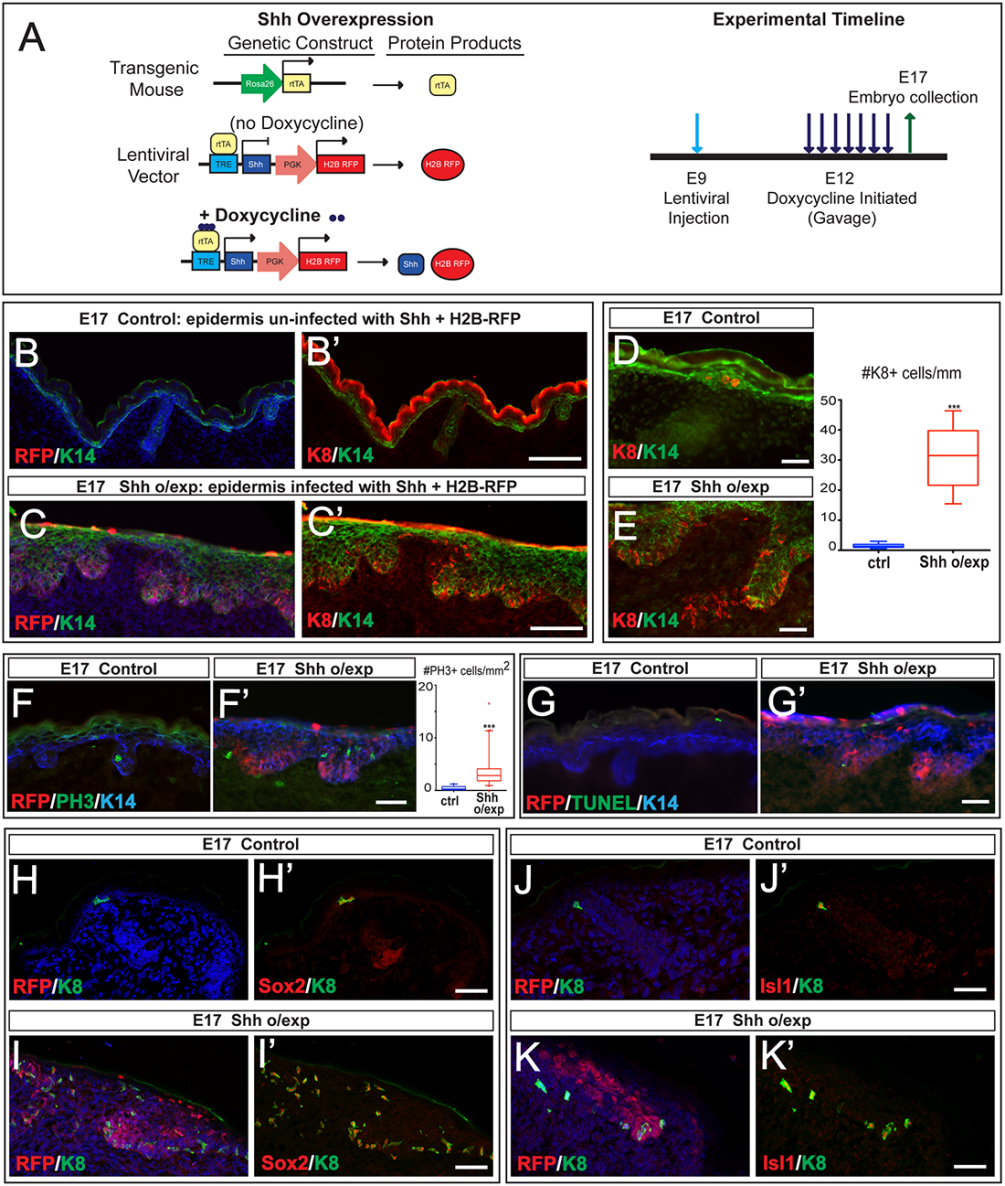
Shh overexpression results in increased formation of cells expressing Merkel markers. (A) Schematic diagram showing transgenic mouse and lentiviral constructs for the Shh overexpression experiment. CD1 female mice were mated with male Rosa26-rtTA mice and *in utero* lentiviral injections were performed in pregnant females E9. Doxycycline treatment was initiated by gavage at E12, and females were continuously fed Doxycycline until embryo collection at E17. In the presence of Doxycycline, lentivirus-injected mouse embryos express both H2B-RFP and Shh proteins. (B-E) IF stainings showing the altered morphology of epidermis infected with Shh+H2B-RFP (C,C’) and the significant increase in the numbers of Krt8(+) (K8) cells in the infected epidermis (C,C’,E) compared to control, un-infected epidermis (E17 control) (B,B’,D). Quantification of Krt8(+) cells per mm of back skin in E17 control and Shh overexpression (right panel) (p<0.0001). (F,F’) IF staining for Phospho-Histone H3 (PH3) showing a significant increase in proliferation in E17 Shh o/exp epidermis (F’) compared to control (F). Quantification of number of PH3(+) per mm^2^ of Krt14(+) epidermal cells is represented on the right (p<0.0001). (G,G’) TUNEL staining showing no increase in apoptosis in E17 Shh o/exp epidermis (G’) compared to control (G). Note that cells undergoing cornification are TUNEL(+), as previously reported [33]. (H-I’) IF staining showing an increase in the number of Sox2(+)/Krt8(+) cells in E17 Shh o/exp epidermis (I,I’) compared to control (H,H’). (J-K’) IF stainings showing an increase in the number of Isl1(+)/Krt8(+) cells in E17 Shh o/exp epidermis (K,K’) compared to control (J,J’). Scale bars: (B-C’): 100μm; (D-K’): 25 μm.

Since intraepidermal Shh signaling is required for Merkel specification, we wanted to investigate whether increased expression of Shh in the developing epidermis results in increased production of Merkel cells. Previous studies have shown that overexpression of Shh in Krt14(+) epidermal progenitors during development results in the formation of Basal cell carcinoma (BCC)-like epidermal hyperplasia and leads to death of the newborn mice [12]. In order to overexpress Shh in the epidermis, we injected high-titer lentiviruses expressing a doxycycline inducible Shh-PGK-H2B-RFP construct into amniotic liquids of E9 Rosa26-rtTA embryos to infect the epidermis (Fig 4A, left). We administered doxycycline to the pregnant females starting at E12 and collected the injected embryos at E17 (Fig 4A, right). Similar to previous reports [12], overexpression of Shh, identified by RFP expression, resulted in BCC-like epidermal hyperplasia, while the control, uninfected epidermis was not altered (Fig 4B and 4C). Consistent with the observed epidermal alterations, the overexpression of Shh resulted in increased proliferation (Fig 4F and 4F’), while there was no significant increase in apoptosis (Fig 4G and 4G’).

Immunofluorescence staining for Merkel cell marker Krt8 revealed that the regions with increased Shh expression had a drastic increase in the number of Krt8(+) cells (Fig 4C, 4C’ and 4E) compared to control regions (Fig 4B, 4B’ and 4D). Shh overexpression resulted in disruption of the Krt14(+) layer (Fig 4E), and, interestingly, the Krt8(+) cells were often localized to these areas (Fig 4E). To determine whether the ectopic Krt8(+) cells were indeed Merkel cells, we performed immunofluorescence stainings for early Merkel cell markers Sox2 (Fig 4H and 4I’) and Isl1 (Fig 4J and 4K’) and found that the ectopic Krt8(+) cells also expressed Sox2 (Fig 4I and 4I’) and Isl1 (Fig 4K and 4K’), suggesting that overexpression of Shh results in the production of ectopic Merkel cells. Together, our data indicate that epidermal Shh signaling promotes Merkel cell specification during development.

### Loss of PRC2 results in ectopic formation of Merkel cells around all hair follicle types

Our data show that epidermal Shh signaling is required for Merkel cell specification. Interestingly, while Merkel cells only form around the primary hairs of the murine dorsal skin, Shh signaling is not unique to primary hairs, as Shh ligand is produced by all types of hair follicles and Shh signaling is globally required for hair follicle down-growth. We therefore aimed to understand how Merkel cell formation is restricted to primary hair follicles.

**Fig 5:**
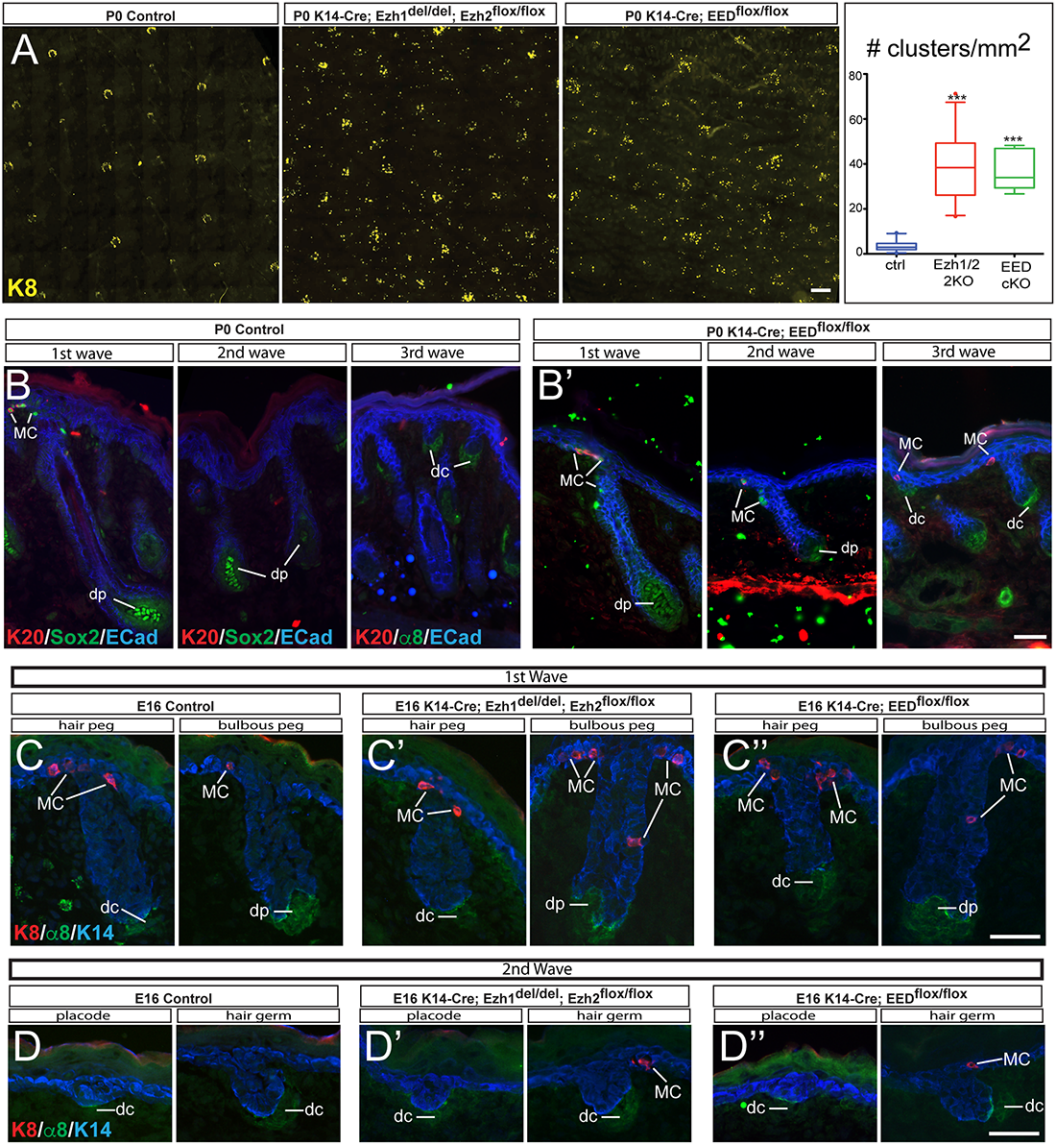
Loss of PRC2 results in ectopic formation of Merkel cells around all hair follicle types. (A) Whole-mount immunofluorescence staining showing Merkel cell-specific marker Krt8 (K8) in P0 Ezh1/2 2KO (K14-Cre; Ezh1^del/del^;Ezh2^flox/flox^) and EED cKO (K14-Cre; EED^flox/flox^) epidermis compared to control (ctrl). Clusters of Merkel cells (≥3 Krt8(+) cells) are quantified, and the number of clusters of Merkel cells per mm^2^ is presented to the right (Kruskal-Wallis test p<0.0001; ctrl vs. Ezh1/2 2KO, *** p<0.0001; ctrl vs. EED cKO, *** p<0.0001). (B-B’) All hair types can have adjacent Merkel cells in EED cKO (B’), while only first wave hair follicles have adjacent Merkel cells in WT (B) epidermis. IF stainings for Sox2 and integrin α8 (α8) are used to label the dermal papillae (dp) or dermal condensate (dc) of different hair follicle types. The dermal papillae of first (left) and second (middle) wave hair follicles are Sox2(+), and the two types of hair follicles can be discriminated by size. The dermal papillae of the third (right) wave hair follicles are Sox2(-)/α8(+), and these hair follicles are very short at P0. IF staining for Sox2 identifies early-specified Merkel Cells (MC) and Krt20 (K20) identifies mature Merkel cells in the epidermis, which is labeled with E-Cadherin (ECad). (C-D”) Early immature Krt8(+) Merkel cells are found around the WT first wave (C) of developing hair follicles, but not the second wave (D) (control: 0% of placodes, 0.81% of hair germs, 19.65% of hair pegs, and 63.89% of bulbous pegs harbor Merkel cells around them; 148 hair follicles analyzed). (C’,C”,D’,D”) Krt8(+) Merkel cells are found around second wave and first wave developing hair follicles in Ezh1/2 2KO (C’,D’) and EED cKO (C”,D”) E16 embryos (Ezh1/2 2KO: 11.26% of placodes, 23.64% of hair germs, 58.33% of hair pegs, and 100% of bulbous pegs harbor Merkel cells around them; 160 hair follicles analyzed. EED cKO: 12.04% of placodes, 25.76% of hair germs, 70.37% of hair pegs, and 100% of bulbous pegs harbor Merkel cells around them; 213 hair follicles analyzed). Krt14 (K14) labels the developing hair follicles, and integrin α8 labels the dermal condensate in the first stages of hair follicle development; this becomes the dermal papilla in the bulbous peg stage. At E16, the first wave hair follicles are in the hair peg or bulbous peg stages (C-C”), while the second wave hair follicles are in the placode and hair germ stages (D-D”). Scale bars (A): 200 μm; (B-D”) 25 μm.

Our previous studies have shown that when any essential PRC2 subunits are conditionally ablated from the epidermis, there is an increase in the number of Merkel cells [29,30]. To further analyze the role of PRC2 in Merkel cell development, we further analyzed skin-conditional knockouts for either the histone methyltransferases Ezh1 and Ezh2 (Ezh1/2 2KO) or EED (EED cKO). We performed whole-mount immunofluorescence analysis of the Ezh1/2-null and EED-null back skin and, surprisingly, found that the ectopic Krt8(+) Merkel cells were not uniformly distributed in the knockout epidermis. Instead, Ezh1/2-null and EED-null ectopic Merkel cells were clustered, and we observed an increase in the number of these clusters in Ezh1/2-null epidermis (Fig 5A). The particular patterning of the PRC2-null Merkel cell clusters (Fig 5A) led us to hypothesize that the ectopic Merkel cells in the PRC2-null epidermis are associated with hair follicles and, importantly, with different types of hair follicles. We have previously shown that hair formation is not affected in neonate Ezh1/2-null or EED-null skin, with the hair follicles being specified in normal numbers and with correct developmental timing (S5A Fig) [29,30,45]. Thus, we investigated whether PRC2-null ectopic Merkel cells are indeed associated with different types of hair follicles.

While different types of hair follicles vary in structure, there are no known molecular markers to distinguish between them. Interestingly, recent studies have shown that the dermal papilla cells differ between some hair types [46–48]. For example, the transcription factor Sox2 is absent from the dermal papillae of tertiary hairs, but is present in the dermal papillae of the primary and secondary hair follicles [46,47]. The different hair follicle types also differ in their developmental timing: primary hairs, or first wave follicles, are the first hairs to appear in the back skin at E14, secondary hairs are formed during the second wave of hair follicle development starting at E16, and tertiary hair follicles are formed during the third wave, appearing just before birth (Fig 1A) [2,31,32]. Thus, we were able to identify the different hair types by their developmental timing, as well as co-staining for Sox2 and integrin α8 (α8), a general dermal papilla marker [47,48]. In newborn mice, the 1^st^ wave hairs are Sox2(+)/α8(+), contain all differentiated layers, and are the longest follicles in skin; the 2^nd^ wave hairs are Sox2(+)/α8(+), but are not yet fully formed, and are still growing downwards; the 3^rd^ wave hairs are Sox2(-)/α8(+), small, and just starting to grow downwards (Fig 5B). Consistent with previous reports, we confirmed that in the control dorsal skin, Krt20(+)/Sox2(+) Merkel cells only appear around the primary hairs and not the other hair types (Fig 5B) [2]. Intriguingly, in PRC2-null epidermis, we identified Merkel cells around both 2^nd^ wave and 3^rd^ wave hair follicles (Figs 5B’ and S5B), indicating that the ectopic Merkel cells can be found around all hair types in PRC2-null skin. We concluded that the loss of PRC2 repression results in the formation of ectopic Merkel cells that are associated with all hair follicle types.

Next, we wanted to determine whether the formation of PRC2-null ectopic Merkel cells is also linked to hair follicle development, as with control Merkel cells. To do this, we analyzed Ezh1/2-null and EED-null skins at E16, as this time point catches follicles of different waves at different developmental stages. For both control and PRC2-null skins at E16, the first wave hair follicles were at hair peg or bulbous peg stages, the second wave follicles were at placode or hair germ stages, and the third wave follicles were yet to be formed (Figs 1A and 5C and 5D”) [34]. Immunofluorescence staining for early Merkel cell marker Krt8 revealed that, not surprisingly, the first wave hair follicles in both control and PRC2-null skin contained Merkel cells (Fig 5C–5C”), while control second wave follicles completely lacked Merkel cells (Fig 5D). Importantly, many PRC2-null second wave hair follicles contained Merkel cells (Fig 5D’ and 5D”). PRC2-null Merkel cells were associated with hair follicles at the hair germ stage, and even some at the placode stage (Fig 5D’ and 5D”), but they were not detected in the epidermis between hair follicles. Finally, no alterations in proliferation were observed in PRC2-null hair follicles or IFE at E16 (S5C Fig). Taken together, these data indicate that loss of PRC2 results in the formation of ectopic Merkel cells that are associated with all types of hair follicles, and, similar to WT Merkel cells, the development of PRC2-null Merkel cells coincides with the early stages of hair follicle formation.

### PRC2 regulates the establishment of a competent field for Merkel cell specification that requires Shh inductive signaling to produce Merkel cells

**Fig 6:**
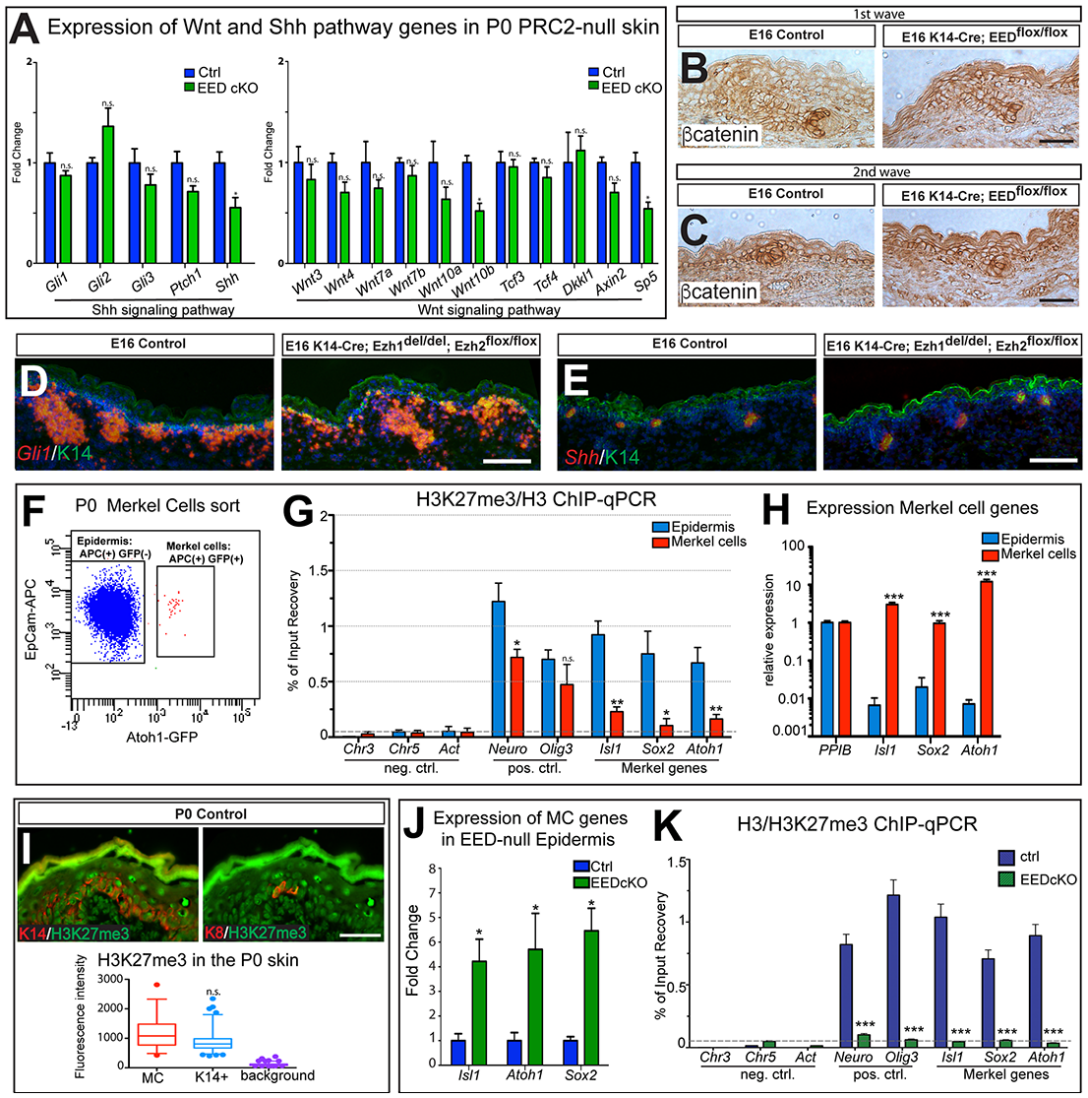
Loss of EED does not affect the hair follicle microenvironment, but leads to upregulation of Merkel cell differentiation genes. (A-E) Shh and Wnt pathways do not appear to be majorly altered in PRC-null developing skin. (A) RT-qPCR analysis of Shh pathway genes shows no significant difference in their expression in P0 EED cKO (K14-Cre; EED^flox/flox^) compared to control epidermis, while *Shh* expression is slightly reduced in EED-null compared to control epidermis (Gli1, p = 0.2000; Gli2, p = 0.1143; Gli3, p = 0.3429; Ptch1, p = 0.1143; Shh, p = 0.0286). RT-qPCR analysis of Wnt pathway genes shows no significant difference in expression of most genes in P0 EED cKO compared to control epidermis (Wnt3, p = 0.4857; Wnt4, p = 0.1143; Wnt7a, p = 0.3429; Wnt7b, p = 0.4857; Wnt10a, p = 0.2000; Wnt10b, p = 0.0286; Tcf3, p = 0.6857; Tcf4, p = 0.4857; Dkkl1, p = 0.6857; Axin2, p = 0.2000; Sp5, p = 0.0286). (B,C) Immunohistochemistry staining for β-catenin does not show major differences in expression or nuclear staining in first wave (B) or second wave (C) hair follicles in EED cKO skin compared to control at E16. Note that, as has been previously described, the stratum corneum is prematurely acquired in the PRC2-null E16 embryo [51]. (D,E) *In situ* hybridization for *Gli1* RNA (D) and *Shh* RNA (E) shows no major changes in expression in Ezh1/2 2KO (K14-Cre; Ezh1^del/del^;Ezh2^flox/flox^) skin compared to control at E16. (F) FACS scheme for Merkel cell (MC) sorting. After gating on singlets and live cells, EpCAM-APC staining was used to gate on all epidermal cells and Atoh1-GFP labels Merkel cells. EpCAM-APC(+) Atoh1-GFP(-) cells were sorted as epidermal controls. (G) ChIP-qPCR showing significantly lower levels of H3K27me3 at Merkel genes, *Isl1*, *Sox2*, and *Atoh1*, in FACS-sorted Merkel cells compared to FACS-sorted epidermal cells. (Neuro, p = 0.0411; Olig1, p = 0.0200; Isl1, p = 0.0022; Sox2, p = 0.0194; Atoh1, p = 0.0050). (H) RT-qPCR showing specific expression of MC signature genes *Isl1*, *Sox2*, and *Atoh1* in FACS-sorted Merkel cells compared to FACS-sorted epidermal cells (*Isl1*, p = 0.0004; *Sox2*, p = 0.0004; *Atoh1*, p = 0.0004) (I) IF staining showing that Krt8(+) (K8) MCs have the H3K27me3 mark in P0 control Krt14(+) (K14) epidermis. Krt14(+) cells serve as a positive control for H3K27me3 staining. Quantification of H3K27me3 staining intensity (below) (Kruskal-Wallis test p<0.0001; MC vs. K14(+), n.s. p>0.05). (J) RT-qPCR in skin epidermal samples showing upregulation of Merkel cell genes *Isl1*, *Atoh1* and *Sox2* in EED cKO animals compared to control. The average of at least three animals is presented compared to Cre(-) siblings (WT) *(Isl1*, p = 0.0286; *Atoh1*, p = 0.0286; *Sox2*, p = 0.0286). (K) ChIP-qPCR analysis in skin epidermal samples from WT and EED cKO animals showing specific H3K27me3 signal at the *Isl1*, *Sox2*, and *Atoh1* promoters. *Actin* is used as a negative control, and *Neuro* and *Olig3* are used as positive controls. Dotted line shows the level of the negative region (all p<0.0001). Scale bars: (B,C,I): 25 μm; (D,E): 100μm.

As PRC2 functions as a transcriptional repressor [26,49,50], we hypothesized that the increased Merkel cell formation observed in the PRC2-null epidermis could be due to increased Shh signaling activity, resulting in the expansion of the signaling required for Merkel cell formation. Interestingly, RT-qPCR analysis did not reveal derepression of critical components of the Shh signaling pathway or activation of its downstream target genes in neonatal PRC2-null epidermis (Fig 6A, left). *Shh* and *Gli1 in situ* hybridization performed on PRC2-null skins further confirmed the RT-qPCR analysis, indicating that there was no detectable increase in Shh signaling in the developing epidermis (Fig 6D and 6E). Additionally, RT-qPCR analysis for critical components of the Wnt signaling pathway and its downstream targets did not reveal derepression of these genes in the PRC2-null epidermis (Fig 6A, right). Immunohistochemistry staining for β-catenin in PRC2-null skin at E16 indicated that the loss of PRC2 did not result in increased nuclear β-catenin staining in first or second wave hair follicles, or in the IFE, when compared to control skin (Fig 6B and 6C). Finally, expression profiling of the PRC2-null neonate epidermis revealed that the major singling pathways involved in hair follicle development [25] are not affected in EED-null skin epithelium (S6A Fig).

If PRC2 does not alter the hair follicle microenvironment, how does loss of PRC2 result in ectopic Merkel cell formation? To start addressing this question, we first analyzed how PRC2-mediated repression is regulated in Merkel cells. We performed fluorescent activated cell sorting (FACS) and purified Atoh1-GFP(+) Merkel cells and Atoh1-GFP(-) epidermal cells (Fig 6F). We next subjected the isolated cells to chromatin immunoprecipitation assay followed by semi-quantitavie PCR (ChIP-qPCR) analysis to investigate the presence of PRC2-dependent repressive histone mark H3K27me3 at key Merkel cell differentiation genes, *Atoh1*, *Sox2*, and *Isl1* [26,29,43,52,53]. The analysis revealed the presence of H3K27me3 at *Atoh1*, *Isl1*, and *Sox2* in epidermal cells (Fig 6G), where these genes are silenced (Fig 6H). The level of this histone mark at the Merkel-specific genes was significantly reduced in Merkel cells (Fig 6G), where *Atoh1*, *Isl1*, and *Sox2* were expressed (Fig 6H). Interestingly, the reduction in PRC2-mediated repression in Merkel cells was not global. Indeed, while the expression of PRC2 subunits *Ezh2*, *EED*, and *Suz12* was reduced in Merkel cells, compared to the epidermis (S6B Fig), immunofluorescent studies revealed that at global levels, the H3K27me3 mark was not reduced in Merkel cells either during embryonic development, at E16, (S6C Fig) or at birth (Fig 6I). Thus, PRC2-mediated repression is relieved from the *Atoh1*, *Sox2*, and *Isl1* genes in Merkel cells.

Transcriptional profiling of EED-null neonatal epidermal cells revealed that loss of PRC2 results in derepression of *Atoh1*, *Sox2*, and *Isl1* (Figs 6J and S6A). This is in accordance with our previous data, which showed that loss of PRC2 results in derepression of *Sox2* and *Isl1* in the epidermis [29,30]. ChIP-qPCR confirmed the loss of PRC2-dependent H3K27me3 mark in the EED-null epidermis (Fig 6K). Together, these data indicate that loss of PRC2-mediated repression in the epidermis leads to upregulation of key Merkel cell differentiation genes.

**Fig 7:**
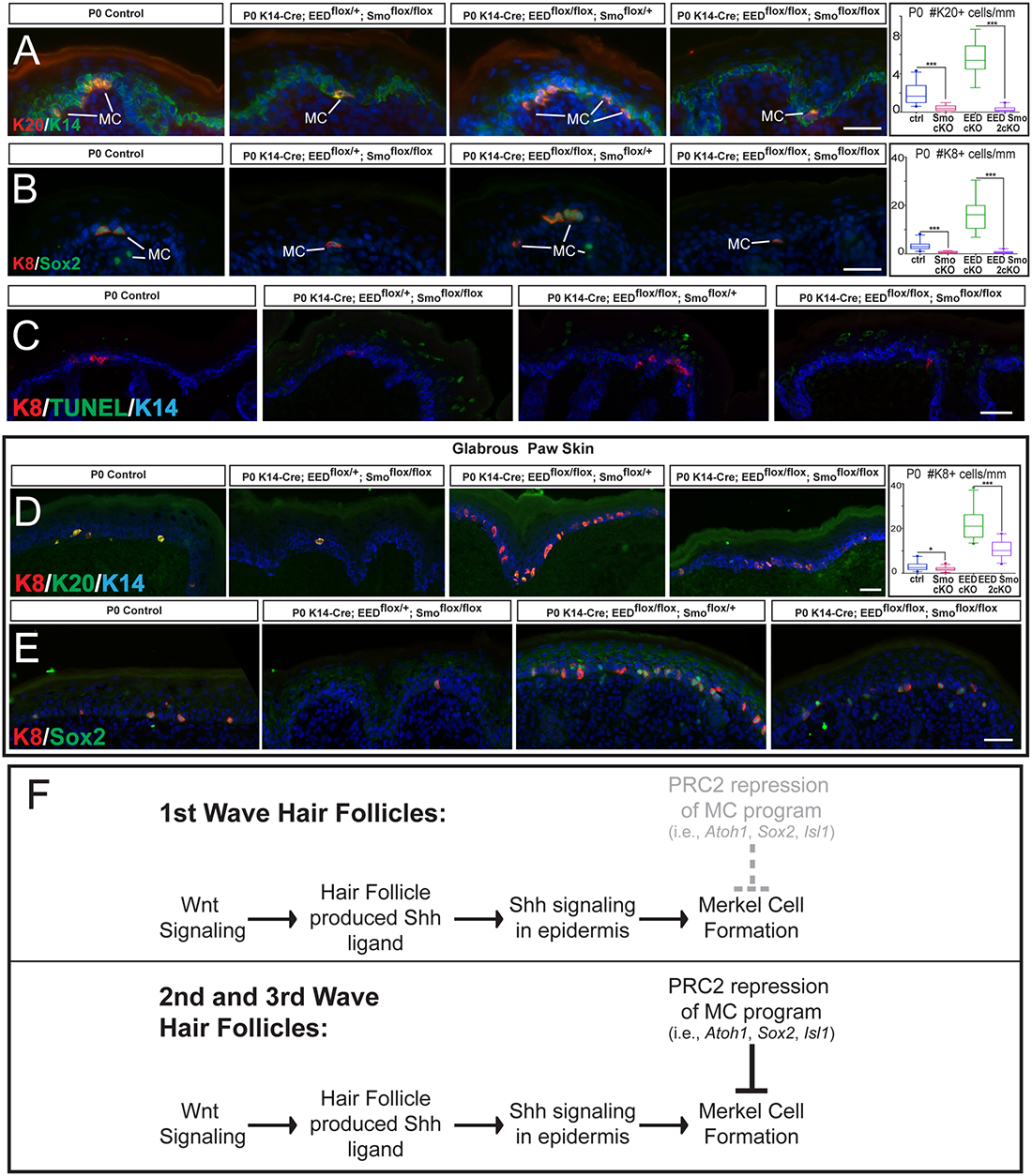
Concurrent loss of PRC2 and Shh signaling in the epidermis results in dramatically reduced numbers of Merkel Cells. (A-B) IF stainings for Merkel cell (MC) markers Krt20 (K20) (A), Krt8 (K8) (B), and Sox2 (B) show a dramatic reduction in the number of Merkel cells in P0 EED Smo epidermis-conditional double knockout (EED Smo 2cKO) (K14-Cre; EED^flox/flox^;Smo^flox/flox^) mice compared to control (ctrl) and EED cKO (K14-Cre; EED^flox/flox^; Smo^flox/+^) mice. Quantification of the number of Krt20(+) quantified to the right of A (ctrl vs. Smo cKO, p<0.0001; EED cKO vs. EED Smo 2cKO, p<0.0001). Quantification of number of Krt8(+) quantified to the right of B (ctrl vs. Smo cKO, p<0.0001; EED cKO vs. EED Smo 2cKO, p<0.0001). (C) TUNEL staining shows no increase in apoptosis in the Krt14(+) basal layer of P0 EED Smo 2cKO. Note that cells undergoing cornification in the suprabasal layers stain positive for TUNEL, as previously reported [33]. (D,E) IF stainings for Merkel cell markers Krt8 (D, E), Krt20 (D), and Sox2 (E) show that significantly fewer Merkel cells are present in the glabrous paw skin of P0 EED Smo 2cKO mice compared to EED cKO paws. Quantification of Krt8(+) cells (right panel of D) (ctrl vs. Smo cKO, p = 0.0317; EED cKO vs. EED Smo 2cKO, p<0.0001). (F) Schematic diagram of the signaling events regulating Merkel cell specification in first wave of hair follicle development. Hair follicle dependent intraepidermal signaling promotes Merkel cell specification. These signaling events also occur during second and third wave of hair follicle morphogenesis but these hair follicles do not harbor Merkel cells due to PRC2-mediated repression of Merkel genes. Scale bars: (A-E): 25 μm.

These findings led us to hypothesize that PRC2 regulates the competent field of epidermal cells capable to differentiate into Merkel cells, but which requires inductive Shh signaling to fully differentiate into Merkel cells. To test this hypothesis, we generated EED Smo epidermis-conditional double-knockout mice (EED Smo 2cKO). If PRC2 regulates the competence of the epidermis to produce Merkel cells in response to Shh-dependent inductive signals, we predicted that the EED Smo 2cKO would have a significant reduction in Merkel cell specification. Indeed, neonate EED Smo 2cKO mice exhibited a highly significant decrease in the number of Merkel cells formed when compared to control and EED cKO epidermis, as in the Smo cKO (Fig 7A and 7B). There were no significant alterations in proliferation or apoptosis in the EED Smo 2cKO mice (Figs 7C, S7A and S7B).

Finally, we analyzed the glabrous paw epidermis of EED Smo 2cKO mice. We observed that, while there are more Merkel cells in the EED Smo 2cKO mice than in the control and Smo cKO mice, there is a highly significant reduction in the number of Merkel cells when compared to EED cKO paws, which exhibit a dramatic increase in the number of Merkel cells (Fig 7D and 7E). This significant reduction in the number of Merkel cells in the EED Smo 2cKO paw epidermis was not due to increased apoptosis (S7C Fig).

Together, these data indicate that the loss of PRC2 leads to derepression of critical Merkel cell differentiation transcription factors and results in the expansion of the competent field of cells capable of producing Merkel cells. In the dorsal skin, ectopic Merkel cells arise only around hair follicles, which provide the local Shh signaling necessary for Merkel cell formation. While specification of Merkel cells in the glabrous paw epidermis has not yet been well characterized, intraepidermal Shh does appear to play a similar role in Merkel cell specification in this region as well (Fig 7F).

## Discussion

In this paper, we set out to determine the molecular mechanisms controlling the development of Merkel cells, mechanoreceptor cells involved in touch sensations. Our data show that in hairy skin, the primary hair follicles function as an essential niche required for Merkel cell formation. We show that defects in hair follicle development in β-Catenin cKO and Shh KO mice result in a complete absence of Merkel cells. Furthermore, we found that intraepidermal Shh signaling, initiated by the production of Shh ligand in the developing hairs, is required for Merkel cell specification. The importance of the signals produced by one skin lineage for the specification of another elegantly demonstrates the complex interplay between the developmental processes that assures proper skin patterning.

Since Shh is produced by all developing hairs and Shh signaling is associated with all hair follicle types, it is likely that there are developmentally regulated restrictive signals that function to ensure that the establishment of Merkel cells occurs solely around the primary hairs. Our data show that PRC2-mediated repression performs this function and restricts Merkel cell production to primary hairs. Indeed, loss of PRC2 in the epidermis results in ectopic Merkel cells that are localized around all hair follicle types. We show that loss of PRC2 does not alter hair follicle development [29,30] or the levels of Shh signaling in the skin epithelium. Instead, it leads to derepression of *Sox2*, *Atoh1*, and *Isl1*, which encode critical Merkel cell-specific transcription factors essential for Merkel cell differentiation [26,29,43,52,53]. These data indicate that the loss of PRC2 expands the field of epidermal cells that are capable of differentiating into Merkel cells. Accordingly, concomitant loss of PRC2 and Smo significantly reduces Merkel cell formation in comparison to loss of PRC2 alone, indicating that the enhanced potential to form Merkel cells observed in the PRC2-null skin requires inductive Shh signaling.

How does PRC2 restrict Merkel cell formation exclusively to primary hair follicles? One possibility is that a primary-hair-specific signal relieves PRC2-mediated repression from the Merkel cell differentiation genes, allowing for Merkel cell formation. It has been shown that abrogation of Eda/Edar signaling or Fgf20 in the skin specifically disrupts primary hair follicle development, suggesting that Eda/Edar- and/or Fgf20-dependent pathways could play a role in this process [54–57]. Although disruption of Eda/Edar signaling has been shown to result in Merkel cell loss [28], loss of expression of Merkel cell markers observed in Edar loss of function can be rescued by activation of Shh signaling (personal communication, I. Brownell), suggesting that Eda/Edar only regulates Merkel cell formation indirectly by activating Shh signaling. Additionally, Merkel cell formation is not significantly affected in mice that lack Fgf20 (personal communication, I. Brownell). Furthermore, while it has been proposed that the dermal papillae of different hair follicles are intrinsically different [58], recent studies have suggested that the hair types are not intrinsically different and can switch in the adult, in association with changes in the number of cells in the dermal papilla [59,60]. Together, these data suggest that a primary-hair-specific signal is not likely to be involved in relieving PRC2-mediated repression during development.

Alternatively, as the primary hair follicles are the first follicles to form, their unique association with Merkel cells might be due, instead, to temporal signals that modulate Polycomb repression during embryogenesis. As primary hair follicles form around E14, it is possible that PRC2-mediated repression of the Merkel cell differentiation program is more promiscuous at this time point, whereas at E16 and E18, when other hair types are formed, PRC2 repression is strong and represses the Merkel cell differentiation genes. The PRC2 complex has been shown to interact with the PRC1 complex, DNA methylation enzymes, and other repressive chromatin regulators [49,61]. Thus, PRC2 might recruit additional repressive complexes to Merkel cell differentiation genes at later developmental stages and lock them in the constrictively silenced state. The mechanisms controlling PRC2 activity in epidermal progenitors during development will be an exciting area of future investigation.

The identified role of PRC2 in repressing a differentiation program in epidermal progenitors is somewhat surprising, as the majority of studies of somatic stem cells have shown that PRC2 promotes proliferation and survival [49,61,62]. In embryonic stem cells, PRC2 has been shown to repress key differentiation genes; however, in contrast to our findings in epidermal progenitors, PRC2-null embryonic stem cells maintain their pluripotent state and do not undergo differentiation [49,61,63]. We show that PRC2 restricts the Merkel cell program in epidermal progenitors by targeting and repressing *Atoh1*, *Sox2*, and *Isl1* genes. Similar to what we find in epidermal progenitors, the *Atoh1* gene is targeted by the PRC2-dependent H3K27me3 mark in prosensory domain progenitors, which give rise to inner ear cells. Importantly, upon differentiation of these progenitors in nascent hair cells, there is a reduction of H3K27me3 at the *Atoh1* locus, which accompanies upregulation of *Atoh1* expression [26]. In this regard, it will be interesting to evaluate the role for PRC2 in controlling inner ear formation and to determine whether loss of PRC2 leads to upregulation of *Atoh1* expression and, possibly, ectopic hair cells.

Our studies showing that Merkel cell specification requires the developing hair follicles, which provide necessary inductive signals, illustrates the essential function of the hair follicle as a niche for Merkel cell formation. Stem cell niches have long been known to play essential roles in stem cell maintenance and survival, and regulation of stem cell activity. Importantly, niche dysfunction has been associated with stem cell aging and cancer [64]. Additional signaling pathways, including BMP and FGF, amongst others, that regulate the growth of the hair follicle [64] might also promote Merkel cell formation. Crosstalk between neurons and Merkel cells should also be further investigated. While innervation of hair follicles occurs at early stages of hair development and coincides with the appearance of Merkel cells [65], it has been shown that Merkel cells only become innervated after birth [27]. Furthermore, neuronal-derived Shh protein is not required for Merkel cell specification during development [66] and touch dome innervation occurs in the absence of Merkel cells [52]. In light of recent data showing that nerve-secreted Shh is required for the self-renewal and maintenance of adult touch domes [66,67], the interdependence of neuronal and Merkel cell morphogenesis should be further investigated.

While most of our studies were performed on murine dorsal skin, our findings on the importance of epidermal Shh signaling in promoting Merkel cell development, and of PRC2 in repressing Merkel cell formation hold true in glabrous skin, such as paw epidermis. Importantly, loss of Smo partially represses the expansion of Merkel cells observed in PRC2-null paws, suggesting that the two signaling events are interacting in similar ways in the glabrous to what we observe in the hairy skin. The identified general role for these molecular pathways in controlling Merkel cell formation might be important for understanding the development and homeostasis of Merkel cells in human skin, where Merkel cells are present in the interfollicular and glabrous epidermis, as well as in the outer root sheath of hair follicles [68]. Our data also show that while the epidermal Shh signaling is clearly important for the formation of Merkel cells in the paw epidermis, loss of Smo only partially reduces the number of Merkel cells in the glabrous skin, suggesting that other signaling pathways also regulate Merkel cell specification in this tissue.

Finally, understanding Merkel cell development and how this process is regulated by the local microenvironment might shed light on the mechanisms of the formation of Merkel cell carcinoma, a deadly disease for which there is no effective treatment [69]. Understanding how Merkel cell formation is regulated at a molecular level can, in the future, contribute to therapeutic strategies to treat Merkel cell carcinoma.

## Materials and Methods

### Ethics statement

All mice were housed and cared for according to Icahn School of Medicine at Mount Sinai (ISMMS)- and Institutional Animal Care and Use Committee (IACUC)-approved protocol LA11-0020. Dr. Millar and Dr. Cohen from The Center for Comparative Medicine and Surgery (CCMS) oversee all animal care at the ISMMS. For research purposes and in cases of veterinarian-monitored illness, we use carbon dioxide in accordance with the Panel on Euthanasia of the American Veterinary Medical Association to euthanize animals.

### Mice

All mice were housed and cared for according to MSSM and IACUC approved protocols. Ezh1^deleted^ and Ezh2^flox^ mice were previously reported [45]. EED^flox^ mice were provided by Weipeng Mu and Terry Magnuson [70]. Tcf/Lef:H2B-GFP mice were provided by Anna-Katerina Hadjantonakis [36]. Gli1^LacZ^, Ctnnb1^flox^, Smo^flox^, Shh^EGFPCre^, Atoh1-GFP, R26-rtTA, and Krt14-Cre mice were obtained from The Jackson Laboratory. Wild type C57BL/6 mice were obtained from Charles River Laboratories. Mice were genotyped by PCR using DNA extracted from tail skin. BrdU was administered as previously reported [51]. Briefly, BrdU was administered (50μg BrdU per 1g mouse weight) to mice or pregnant females 3–5 h before sacrificing [45].

### Immunofluorescence and immunohistochemistry

For immunofluorescence, tissues were collected from mice, embedded fresh into OCT, and subsequently cut into 10μm sections using a Leica Cryostat. Embryos collected after lentiviral infection for the Shh overexpression experiment were pre-fixed for in 4% PFA 1h at RT. Slides were fixed for 10 min (or 7 min for slides of Shh overexpression embryos) in 4% PFA and blocked for 1h or overnight in PBS-Triton with BSA/NDS. Primary antibodies were diluted in blocking solution and incubations were carried out for 1h or overnight, followed by incubation in secondary antibodies for 1h at room temperature. Slides were then counterstained with DAPI and mounted using antifade mounting media. Whole-mount immunofluorescence was performed as previously described [26]. Briefly, back skins were collected from newborn mice and placed in Dispase for 1h at 37°C, after which the epidermal portion was peeled from the dermis and fixed in 4% PFA for 2h. Skins were blocked overnight in PBS-Triton with BSA/NDS. Primary antibodies were diluted in blocking solution and incubations were carried out for 4h at room temperature, followed by incubation in secondary antibodies for 4h at room temperature. Skins were then counterstained with DAPI and mounted in antifade mounting media for imaging.

TUNEL stainings were performed using the Roche TUNEL kit (Roche, 11684795910) according to the manufacturer’s instructions.

β-Catenin immunohistochemistry was performed as previously described [71]. Briefly, after rehydration, paraffin-embedded tissues were subjected to antigen retrieval with 10mM citrate buffer pH 6.0 and then incubated overnight at 4°C with primary antibodies, using the M.O.M. kit (Vector Laboratories). Secondary anti-mouse coupled to HRP (1:100) (Vectastain ABC Kit Mouse IgG, Vector Laboratories PK-4002) was used for 1h at room temperature, and the staining was developed with the DAB Peroxidase Substrate Kit (Vector Laboratories SK-4100).

### Detection of β-Galactosidase activity and *in situ* hybridization

Tissues were collected from mice, embedded fresh into OCT, and subsequently cut into 10μm sections using a Leica Cryostat. For detection of β–Galactosidase activity, slides were fixed for 10 min in 4% PFA, washed with PBS, and incubated with 1 mg/ml X-gal substrates in PBS with 100mM NaPO_4_ 1.3 mM MgCl_2_, 3 mM K_3_Fe(CN)_6_, and 3 mM K_4_Fe(CN)_6_ overnight at 37°C. *In situ* hybridization for *Shh* and *Gli1* was performed using RNAscope probes and assays, according to the manufacturer’s instructions (Advanced Cell Diagnostics).

### Microscopy and quantification

Slides were imaged using a Leica DM6000 slide microscope and either 10x, 20x, or 40x objectives. Confocal microscopy was performed using a Leica SP5 DM and either 20x or 40x objectives. The quantification of Merkel cells per mm of skin was performed as described [29]. Briefly, the length of each section was measured and the number of positively stained cells was counted. Typical section lengths were between 7–14 mm. We counted a large number of Merkel cells in the controls conditions (>300 Krt8(+) cells) and then counted the number of Merkel cells in a similar length of skin for the each mutant line. Typically, at least 100 mm of skin were counted for each condition. The quantification of Merkel cell clusters was done using Leica LAS AF software; tile scan images of the dorsal skin where acquired and the number of clusters of ≥3 Krt8(+) cells per mm^2^ was quantified for different areas of the dorsal skin of at least 3 different individuals. Merkel cells in glabrous paw skins were quantified using Leica LAS AF software; 10x tile scan images of sections of the paws where acquired. The number of Merkel cells was quantified on the zoomed-in images and the length of the Krt14(+) epidermis was measured on the large-scale tiled images, using the Leica LAS AF software and ImageJ software. PH3(+) cells were quantified using a fluorescence microscope. The length of each section was measured and the number of positively stained cells was counted. Whenever it was ambiguous whether a PH3(+) cells was in the epidermis, an image with Krt14 counter staining was acquired. For the Shh overexpression experiment, PH3(+) cells where quantified using Leica LAS AF software; the number of PH3(+) cells were quantified on images and the area of Krt14(+) epidermis was measured using ImageJ software. BrdU(+) cells where quantified using Leica LAS AF software; nuclear DAPI staining was used to quantify the total number of cells and the % of BrdU(+) cells was calculated. *Gli1(+)* cells were quantified in the same manner. Fluorescence intensity was calculated from at least three raw, single-channel greyscale images per condition using Leica LAS AF software. Staining in different skin cell populations was compared to background levels of fluorescence, which were measured as non-nuclear areas of the skin and areas outside of the skin in the same image.

### Statistics

In all column bar graphs, mean value ± one standard deviation is presented. Box-and-whisker plots show first to third quartiles around the median, with whiskers showing 5%-95% range and outliers presented as individual data points. All quantifications were performed on multiple cell populations from different animals. To determine the significance between control and EED cKO in the ChIP-qPCR experiment in Fig 6K, an unpaired t-test was used. To determine the significance between two groups in all other experiments, the Mann-Whitney test was performed (GraphPad Prism 5). To determine the significance between more than two compared groups, the Kruskal-Wallis test was performed with the Dunn’s Multiple Comparisons post test (GraphPad Prism 5). For all statistical tests, the p<0.05 level of confidence was accepted for statistical significance, and actual p-values (to four decimal places) were provided in the figure legends.

### Antibodies

Antibodies were used as follows: Krt14 (generous gift of Julie Segre, National Human Genome Research Institute, MD, USA, 1:20,000); Krt8 (Developmental Studies Hybridoma Bank, TROMA-1, 1:500); Krt18 (abcam, ab668, 1:100); Krt20 (Dako, M7019, 1:70); Sox2 (Stemgent, 09–0024, 1:150); Isl1 (abcam, ab109517, 1:250); GFP (abcam, ab13970, 1/1000); Krt5 (generous gift of Elaine Fuchs, The Rockefeller University, NY, USA, 1:500); Phospho-histone H3 (Upstate, 06–570, 1/1000); BrdU (abcam, ab1893, 1:250); Integrin α8 (Santa Cruz, sc-30982, 1:100); Activated Caspase 3 (R&D, AF835, 1:250); β-Catenin (BD Biosciences, 610153, 1:100); H3K27me3 (Millipore, 07–449, 1:300). For IF, secondary Abs coupled to Alexa 488, 549 or 649, were from Jackson Laboratories (1:1000); E-cadherin (Invitrogen, 131900, 1/2000). For immunohistochemistry, secondary anti-mouse coupled to HRP was used (Vectastain ABC Kit Mouse IgG, Vector Laboratories PK-4002).

### Merkel cell sort purification

Newborn back skins from Atoh1-GFP mice were dissected and incubated overnight in 1.26U/ml dispase (Invitrogen) at 4°C. The epidermis was gently peeled from the dermis and incubated for 15 min with 0.25% Trypsin, 2.21 mM EDTA (CORNING). Epidermal cells were washed with PBS and stained with 1:400 EpCAM-APC Antibody (BioLeged 118214) at room temperature for 15 min. Merkel cells were sorted at low pressure using the ARIA sorter (BD) as Atoh1-GFP(+) EpCAM(+). As a control, EpCAM(+) GFP(-) epidermal cells were collected. DAPI was used as a live/dead exclusion dye.

### RNA extraction and analysis

Newborn back skins were incubated overnight in 1.26U/ml dispase (Invitrogen) at 4°C. The epidermis was gently peeled from the dermis and washed with phosphate-buffered saline (PBS). Epidermal cells were dissociated by treatment with 0.25% Trypsin, 2.21 mM EDTA (Corning) for 15 minutes, and dissociated keratinocytes were washed with PBS and lysed in RLT Plus buffer (QIAGEN). FACS purified Merkel and epidermal cells were collected in RLT Plus buffer (QIAGEN). RNA was purified with the RNeasy Plus mini Kit (QIAGEN) according to the manufacturer's instructions. For semi-quantitative analysis, reverse transcription was performed using qScript (Quanta) Superscript Supermix, and qPCR was performed using Roche SYBR green reagents and a Lightcycler480 machine. All primers are listed in S1 Table.

### Two-color microarray-based gene expression analysis

RNA samples were amplified and labeled using the Low Input Quick Amp Labeling Kit (Agilent Technologies, USA) according to manufacturer's instructions. In brief, 100ng of total RNA from each sample was used to prepare Cyanine-3 (Cy3)-labeled cRNA for hybridization. The Universal Mouse Reference RNA (Agilent Technologies) was Cyanine-5 (Cy5)-labeled and used as an internal control, with dye swaps between samples and reference. The RNA Spike-In kit (Agilent Technologies) was used as an external control to monitor the microarray workflow and accuracy.

For microarray hybridization, 300ng of labeled sample was fragmented and hybridized against 300ng of labeled Universal Mouse Reference RNA on SurePrint G3 Mouse GE 8X60K microarrays (Agilent) for 17 hours at 65°C in a rotating hybridization oven (Agilent). Following hybridization, microarrays were washed with GE wash buffer 1 (Agilent) for 1 min at room temperature and with GE wash buffer 2 (Agilent) for 1 min at 37°C. Microarrays were scanned using a SureScan Microarray Scanner (Agilent Technologies). The scanned images were analyzed with Agilent Feature Extraction v12.0.1.1 and GeneSpring v13.0 software (Agilent Technologies). Statistical analysis was carried out using an unpaired t-test and genes with a p-value <0.05 and an absolute fold change ≥2 were considered significantly differentially expressed.

For the construction of the heatmaps, Log2 normalized expression values generated by the GeneSpring software were used for control and EED cKO samples. Fold changes were then generated between the control and EED cKO average expression levels to demonstrate differences in the expression values between the two conditions. Known genes of signaling pathways involved in hair follicle morphogenesis and skin development (Wnt, Shh, FGF, BMP, and Notch signaling) [72], as well as Merkel cell-specific genes were mined from the KEGG database. Expression values for these genes were selected from the data and used for the construction of the heatmaps. Individual heatmaps were built with the R package ggplot2.

The microarray data for this publication were deposited in NCBI’s Gene Expression Omnibus [73] and can be viewed using GEO Series accession number GSE83244 (http://www.ncbi.nlm.nih.gov/geo/query/acc.cgi?acc=GSE83244).

### Chromatin immunoprecipitation

ChIP assays were performed as described [74]. Merkel cells and epidermal control cells were obtained by FACS purification and total epidermal cells were obtained from P0 back skin after dispase treatment, followed by tripsinization of the epidermis. Cells were cross-linked with 1% formaldehyde/PBS solution for 10 min at room temperature. After fixation was stopped with 125mM Glycine, nuclei were extracted in lysis buffer [50mM HEPES (pH 7.5), 140mM NaCl, 1mM EDTA, 10% glycerol, 0.5% NP-40, 0.25% Triton X100] with protease inhibitors (Complete, Roche). Before ChIP, nuclei were resuspended in sonication buffer [10mM Tris-HCl (pH 8), 200mM NaCl, 1mM EDTA, 0.5mM EGTA, 0.1% Na-deoxycholate, 0.5% N-laurylsarcosine, 1% Triton X100], lysed, and sonicated with Bioruptor (Diagenode, UCD-200) according to a 30x regimen of 30 sec. sonication followed by 30 sec. rest at 2.7°C to solubilize and shear cross-linked DNAs. After centrifugation, the supernatant was incubated overnight at 4°C with 40μl of Dynal Protein G magnetic beads (Invitrogen), which had been pre-incubated with 4μl of the H3 or H3K27me3, antibodies. After ChIP, samples were washed with low salt, high salt, LiCl, and Tris-EDTA buffers for 10 min at 4°C. Cross-linking was reversed by overnight incubation at 65°C, followed by RNase A and proteinase K treatment. Samples were purified with ChIP DNA Clean & Concentrator columns (Zymo Research). All qPCR was performed using Roche SYBR green reagents and a Lightcycler480 machine, and the percentage of input recovery was calculated. For histone marks, the histone modifications signal was calculated based on the bulk histone signal. All primers are listed in S1 Table.

### In utero injections

Ultrasound-guided lentiviral injection procedures have been previously described [75]. In brief, R26-rtTA male mice were mated to CD1 female mice to generate R26-rtTA embryos for Shh lentiviral injection. A high-titer (>10^9^ CFU) inducible Shh overexpression lentiviral construct (LV-TRE-Shh-PGK-H2B-RFP) [76] was used to perform microinjections into the amniotic cavities of E9 embryos. E9 timed pregnant mice were anesthetized with 2.5% isoflurane and 1% oxygen, and then positioned on a mouse platform (Integrated Rail System, VisualSonics). A midline incision was performed, and uterine horns were gently exteriorized through the incision and carefully drawn through a ParafilmH flap in the bottom of a sterilized petri dish. Four to five embryos were injected using a micropipette, with 1 μl of Shh overexpression lentivirus for each embryo, and the uterine horn was reinserted into the peritoneal cavity. The abdominal wall and skin were closed with sutures. Expression of the viral construct was induced at E12 by gavage treatment of 200μl doxycycline (10 mg/ml in sterile water, Sigma-Aldrich) to the mice that were pregnant with the injected pups. The pregnant mice were then fed doxycycline chow (200 mg/kg, Bio-Serv) for 5 days. Shh o/exp and control embryos were collected at E17 and fixed with 4% PFA for 1 h. Fixed embryos were washed five times with PBS and embedded in OCT for further analysis.

### Accession numbers

Microarray data have been deposited into the GEO database with accession number GSE83244.

## Supporting Information

S1 Fig Loss of Wnt/β-catenin signaling abrogates hair follicle development and Merkel specification. (A) Hematoxylin and Eosin staining shows that hair follicles are completely absent in the skin of P0 β-cat cKO (K14-Cre; Ctnnb1^flox/flox^) mice. (B) IF for β-cat showing that β-cat protein is not present in the epidermis (epi) in β-cat cKO mice, but is still present in the dermis (der). (C) IF stainings for Merkel cell markers Krt8 (K8) and Isl1 show a complete absence of Merkel cells in P0 β-cat cKO mice compared to control (ctrl). (D) IF staining for the proliferation marker Phospho-Histone H3 (PH3) shows no change in proliferation in the skin of P0 β-cat cKO mice. Quantification of number of PH3(+) cells in control and β-cat cKO P0 skin (right panel of D) (p = 0.7949). (E) IF staining for Activated Caspase 3 (Casp3) shows no defects in apoptosis in P0 β-cat cKO mice compared to Control. Scale bars: (A): 100μm; (B-E): 25 μm.
(TIF)

S2 Fig Shh signaling is required for Merkel cell formation. (A) *In situ* hybridization for *Shh* RNA shows loss of *Shh* expression in E16 Shh KO (Shh^EGFPcre/EGFPcre^) mice compared to control (ctrl). (B) Hematoxylin and Eosin staining shows that hair follicles are arrested at the placode stage in the skin of E18 Shh KO mice. (C) IF staining for the proliferation marker Phospho-Histone H3 (PH3) shows no defects in proliferation in the skin of E18 Shh KO mice. Quantification of number of PH3(+) cells (right panel of C) (p = 0.8052). (D) IF staining for Activated Caspase 3 (Casp3) shows no alterations in apoptosis in the skin of E18 Shh KO mice compared to control. (E) IF staining for Merkel cell markers Krt8 (K8) and Isl1 shows a complete absence of Merkel cells in E16 Shh KO mice compared to control. (F) IF staining for Activated Caspase 3 (Casp3) shows no alterations in apoptosis in the skin of E16 Shh KO mice compared to control. (G) *In situ* hybridization for *Shh* RNA showing loss of Shh expression in the epidermis of P0 Shh cKO (K14-Cre; Shh^flox/flox^) mice when compared to control. (H) IF staining for Merkel cell markers Krt8 (K8) and Krt18 (K18) shows a complete absence of Merkel cells in P0 Shh cKO mice compared to control. (I) IF staining for the proliferation marker Phospho-Histone H3 (PH3) showing no defects in proliferation in the skin of P0 Shh cKO mice. Quantification of number of PH3(+) (right panel of I) (p = 0.1871). (J) IF staining for Activated Caspase 3 (Casp3) showing no defects in apoptosis in the skin of P0 Shh cKO mice. Scale bars: (A, B, G): 100μm; (C-F, H-J): 25μm.
(TIF)

S3 Fig Shh signaling activity in the epidermis is required for Merkel cell formation. (A) Hematoxylin and Eosin (H&E) staining showing that hair follicles develop abnormally in P0 Smo cKO (K14-Cre; Smo^flox/flox^) mice. (B) IF staining for the proliferation marker Phospho-Histone H3 (PH3) showing no defects in proliferation in the skin of Smo cKO mice compared to control (ctrl). Quantification of the number of PH3(+) (right panel of B) (p = 0.3555). (C) IF staining for Activated Caspase 3 (Casp3) showing no increase in apoptosis in the skin of P0 Smo cKO mice. (D) Hematoxylin and Eosin staining showing that hair follicles develop abnormally in the skin of P9 Smo cKO mice compared to control. (E) TUNEL staining showing no defects in apoptosis in the Krt14(+) cells P9 Smo cKO skin. Note that cells undergoing cornification stain positive for TUNEL, as reported previously [33]. (F) IF staining for Merkel cell marker Krt8 (K8) showing a significantly lower number of Krt8(+) cells in P9 Smo cKO skin compared to control. Quantification of Krt8(+) cells (right panel of F) (p<0.0001). (G-H) IF staining for Activated Caspase 3 (G) and TUNEL staining (H) showing no defects in apoptosis in E15 Smo cKO skin compared to control. (I,I’) X-gal staining in mice expressing Gli1-LacZ showing that, at E15, Gli1 is expressed in the developing hair follicles, as well as in the epidermis and dermis surrounding the hair follicles. (J,J’) *In situ* hybridization for *Gli1* RNA confirming that *Gli1* is expressed in the developing hair follicles, as well as in the epidermis and dermis surrounding the hair follicles at E16. Scale bars: (A, D, I, J): 100μm; (B-C, E-H, I’, J’): 25 μm.
(TIF)

S4 Fig Quantification of Merkel cells in glabrous paw epidermis. (A) Tile-scan images of paw sections used for Merkel cell quantification in the glabrous paw skin. Krt14(+) (K14) cells were used to quantify the length of the skin in mm, and the number of Krt8(+) (K8) Merkel cells per mm of glabrous paw skin was quantified in magnified images. (B) TUNEL staining showing no defects in apoptosis in the paws of P0 Smo cKO mice compared to control tissue. Note that cells undergoing cornification in the suprabasal layers stain positive for TUNEL as reported previously [33]. Scale bars: (A, A’): 100μm; (A and A’ magnified images, B): 25 μm.
(TIF)

S5 Fig Loss of PRC2 results in ectopic formation of Merkel cells around all hair follicle types. (A) Hematoxylin and Eosin staining showing that hair follicle development is unaffected in the neonatal P0 skin of Ezh1/2 2KO (K14-Cre; Ezh1^del/del^;Ezh2^flox/flox^) and EED cKO (K14-Cre; EED^flox/flox^) compared to control (ctrl). (B) All hair types can have adjacent Merkel cells (MC) in P0 Ezh1/2 2KO. IF staining for Sox2 and integrin α8 (α8) is used to label the dermal papillae (dp) of different hair follicle types. The dp of first (left) and second (middle) wave hair follicles is Sox2(+), and the two types of hair follicles can be discriminated by size. The dp of the third (right) wave hair follicles is Sox2(-)/α8(+), and these hair follicles are very short at P0. IF staining for Sox2 identifies early-specified Merkel Cells (MC) and Krt20 (K20) identifies mature Merkel cells in the epidermis, which is labeled with E-Cadherin (ECad). (C) IF staining for BrdU shows no significant change in proliferation in the developing interfollicular epidermis (IFE) and hair follicles (HF) in E16 Ezh1/2 2KO and EED cKO compared to control. Quantification of the percentage of BrdU(+) cells in the interfollicular epidermis and hair follicles of E16 control, Ezh1/2 2KO, and EED cKO is presented on the right (IFE, p = 0.9361; HF, p = 0.5707). Scale bars: (A): 100 μm; (B,C): 25 μm.
(TIF)

S6 Fig Loss of PRC2 does not affect the hair follicle microenvironment. (A) Genes of signaling pathways involved in hair follicle morphogenesis and skin development (Wnt, Shh, FGF, BMP, and Notch signaling) [72], as well as Merkel cell-specific genes were mined from the KEGG database. The expression levels of these genes in P0 control and EED cKO (K14-Cre; EED^flox/flox^) skin epithelium (compared to the Universal Mouse Reference RNA) as well as the fold change between the two conditions are represented in heatmaps. (B) RT-qPCR showing significantly less expression of PRC2 subunits *Ezh2*, *EED*, and *Suz12* in P0 Merkel cells compared to control epidermis (Ezh1, p = 1.0000; Ezh2, p = 0.0011; EED, p = 0.0117; Suz12, p = 0.0041). (C) IF staining showing that Krt8(+) (K8) MCs have the H3K27me3 mark in E16 control Krt14(+) (K14) epidermis. Krt14(+) cells serve as a positive control for H3K27me3 staining. Quantification of H3K27me3 staining intensity (right panel of C) (Kruskal-Wallis test p<0.0001; MC vs K14(+), n.s. p>0.05). Scale bars: (C): 25 μm.
(TIF)

S7 Fig Concurrent loss of PRC2 and Shh signaling in the epidermis results in dramatically reduced numbers of Merkel cells. (A) IF staining for the proliferation marker Phospho-Histone H3 (PH3) showing no alterations in proliferation in the skin of P0 EED Smo 2cKO (K14-Cre; EED^flox/flox^;Smo^flox/flox^) mice compared to control (ctrl) epidermis. Quantification of the number of PH3(+) cells (right panel of A) (p = 0.3517). ((B) IF staining for Activated Caspase 3 (Casp3) showing no defects in apoptosis in the dorsal skin of P0 EED Smo 2cKO mice compared to control or EED cKO (K14-Cre; EED^flox/flox^;Smo^flox/+^) mice. (C) TUNEL staining showing no defects in apoptosis in the glabrous skin of P0 EED Smo 2cKO mice compared to control or EED cKO paws. Note that cells undergoing cornification stain positive for TUNEL as reported previously [33]. Unless otherwise indicated, all epidermis represented is dorsal skin. Scale bars: (A-C): 25 μm.
(TIF)

S1 Table List of primers used in this study. (DOCX)
