# Supplementary figures and images for: Polycomb-Mediated Repression and Sonic Hedgehog Signaling Interact to Regulate Merkel Cell Specification during Skin Development
Source: PLoS Genet. 2016 Jul 14;12(7):e1006151. doi: 10.1371/journal.pgen.1006151 (PMC4944976; doi:10.1371/journal.pgen.1006151)

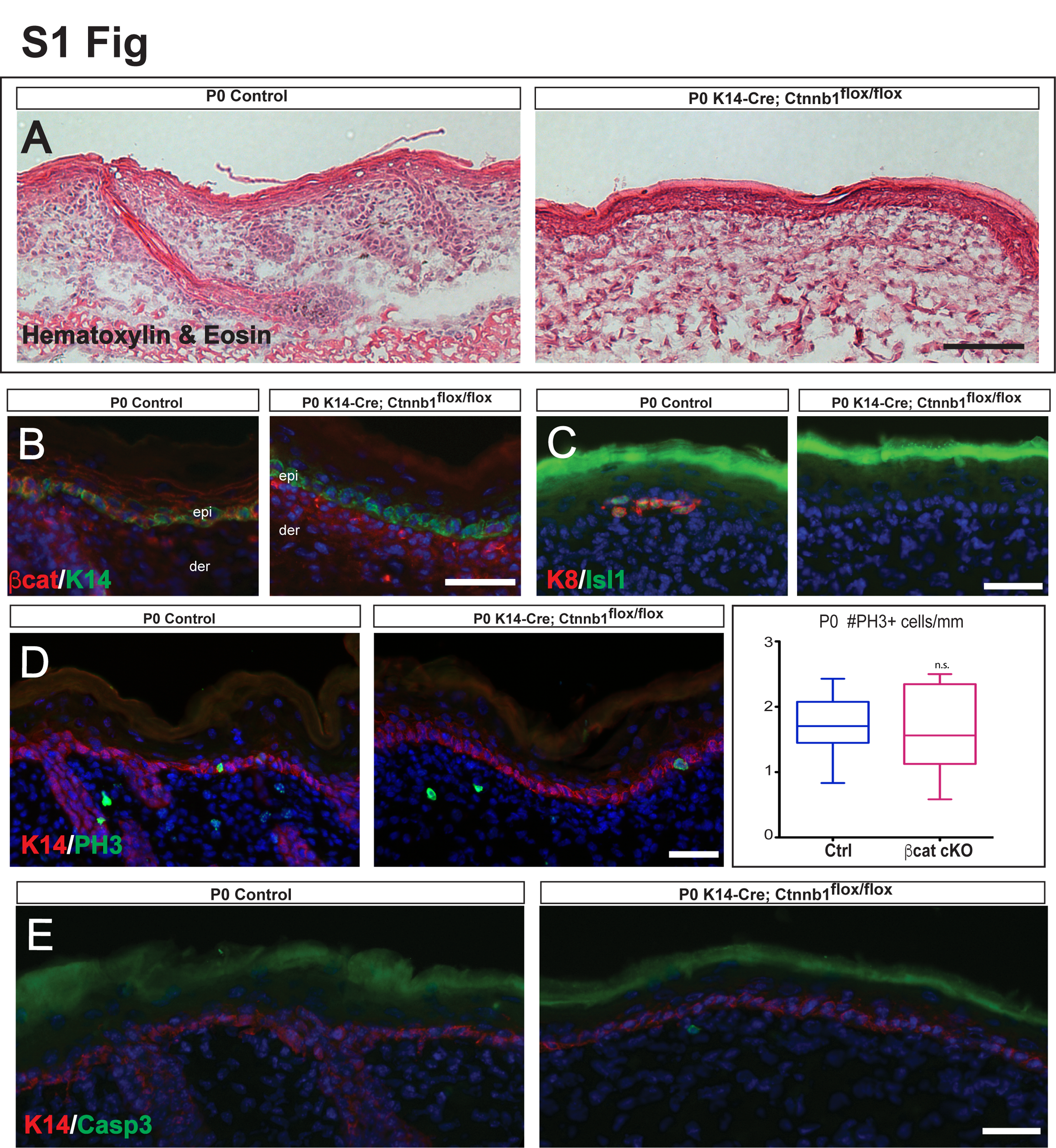

Supplement: S1 Fig — (A) Hematoxylin and Eosin staining shows that hair follicles are completely absent in the skin of P0 β-cat cKO (K14-Cre; Ctnnb1flox/flox) mice. (B) IF for β-cat showing that β-cat protein is not present in the epidermis (epi) in β-cat cKO mice, but is still present in the dermis (der). (C) IF stainings for Merkel cell markers Krt8 (K8) and Isl1 show a complete absence of Merkel cells in P0 β-cat cKO mice compared to control (ctrl). (D) IF staining for the proliferation marker Phospho-Histone H3 (PH3) shows no change in proliferation in the skin of P0 β-cat cKO mice. Quantification of number of PH3(+) cells in control and β-cat cKO P0 skin (right panel of D) (p = 0.7949). (E) IF staining for Activated Caspase 3 (Casp3) shows no defects in apoptosis in P0 β-cat cKO mice compared to Control. Scale bars: (A): 100μm; (B-E): 25 μm. (TIF) [file pgen.1006151.s001.tif]

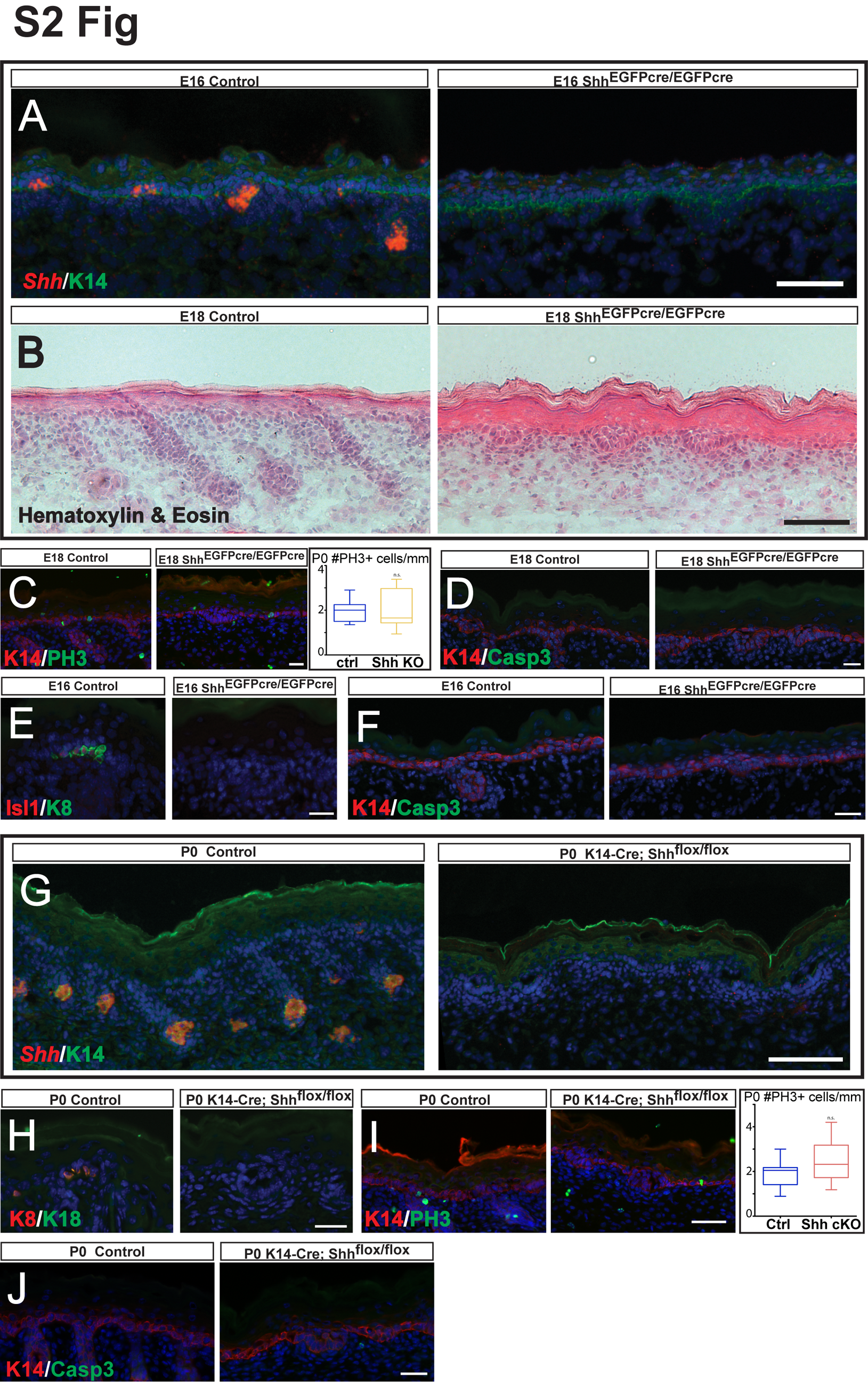

Supplement: S2 Fig — (A) In situ hybridization for Shh RNA shows loss of Shh expression in E16 Shh KO (ShhEGFPcre/EGFPcre) mice compared to control (ctrl). (B) Hematoxylin and Eosin staining shows that hair follicles are arrested at the placode stage in the skin of E18 Shh KO mice. (C) IF staining for the proliferation marker Phospho-Histone H3 (PH3) shows no defects in proliferation in the skin of E18 Shh KO mice. Quantification of number of PH3(+) cells (right panel of C) (p = 0.8052). (D) IF staining for Activated Caspase 3 (Casp3) shows no alterations in apoptosis in the skin of E18 Shh KO mice compared to control. (E) IF staining for Merkel cell markers Krt8 (K8) and Isl1 shows a complete absence of Merkel cells in E16 Shh KO mice compared to control. (F) IF staining for Activated Caspase 3 (Casp3) shows no alterations in apoptosis in the skin of E16 Shh KO mice compared to control. (G) In situ hybridization for Shh RNA showing loss of Shh expression in the epidermis of P0 Shh cKO (K14-Cre; Shhflox/flox) mice when compared to control. (H) IF staining for Merkel cell markers Krt8 (K8) and Krt18 (K18) shows a complete absence of Merkel cells in P0 Shh cKO mice compared to control. (I) IF staining for the proliferation marker Phospho-Histone H3 (PH3) showing no defects in proliferation in the skin of P0 Shh cKO mice. Quantification of number of PH3(+) (right panel of I) (p = 0.1871). (J) IF staining for Activated Caspase 3 (Casp3) showing no defects in apoptosis in the skin of P0 Shh cKO mice. Scale bars: (A, B, G): 100μm; (C-F, H-J): 25μm. (TIF) [file pgen.1006151.s002.tif]

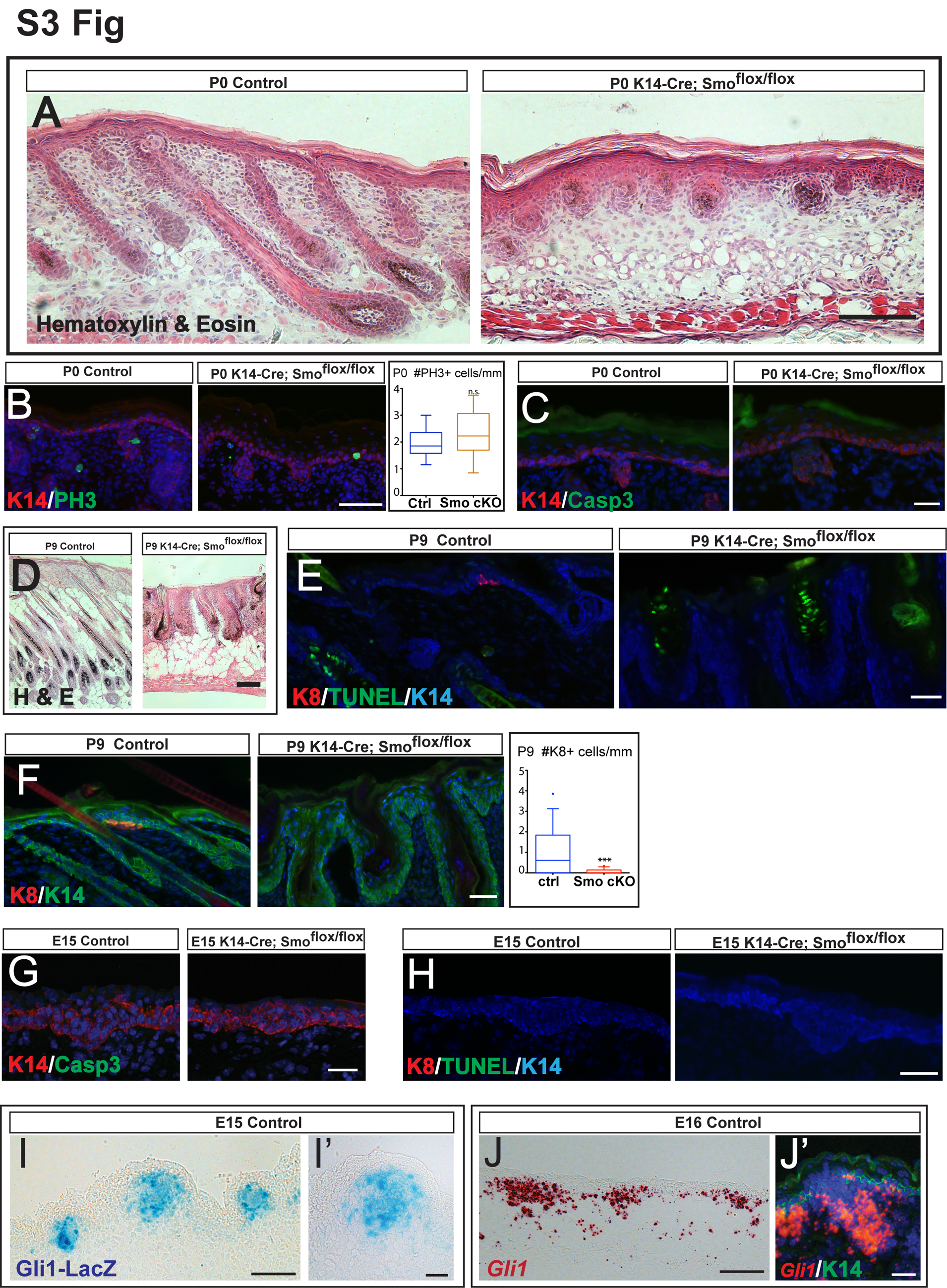

Supplement: S3 Fig — (A) Hematoxylin and Eosin (H&E) staining showing that hair follicles develop abnormally in P0 Smo cKO (K14-Cre; Smoflox/flox) mice. (B) IF staining for the proliferation marker Phospho-Histone H3 (PH3) showing no defects in proliferation in the skin of Smo cKO mice compared to control (ctrl). Quantification of the number of PH3(+) (right panel of B) (p = 0.3555). (C) IF staining for Activated Caspase 3 (Casp3) showing no increase in apoptosis in the skin of P0 Smo cKO mice. (D) Hematoxylin and Eosin staining showing that hair follicles develop abnormally in the skin of P9 Smo cKO mice compared to control. (E) TUNEL staining showing no defects in apoptosis in the Krt14(+) cells P9 Smo cKO skin. Note that cells undergoing cornification stain positive for TUNEL, as reported previously [33]. (F) IF staining for Merkel cell marker Krt8 (K8) showing a significantly lower number of Krt8(+) cells in P9 Smo cKO skin compared to control. Quantification of Krt8(+) cells (right panel of F) (p<0.0001). (G-H) IF staining for Activated Caspase 3 (G) and TUNEL staining (H) showing no defects in apoptosis in E15 Smo cKO skin compared to control. (I,I’) X-gal staining in mice expressing Gli1-LacZ showing that, at E15, Gli1 is expressed in the developing hair follicles, as well as in the epidermis and dermis surrounding the hair follicles. (J,J’) In situ hybridization for Gli1 RNA confirming that Gli1 is expressed in the developing hair follicles, as well as in the epidermis and dermis surrounding the hair follicles at E16. Scale bars: (A, D, I, J): 100μm; (B-C, E-H, I’, J’): 25 μm. (TIF) [file pgen.1006151.s003.tif]

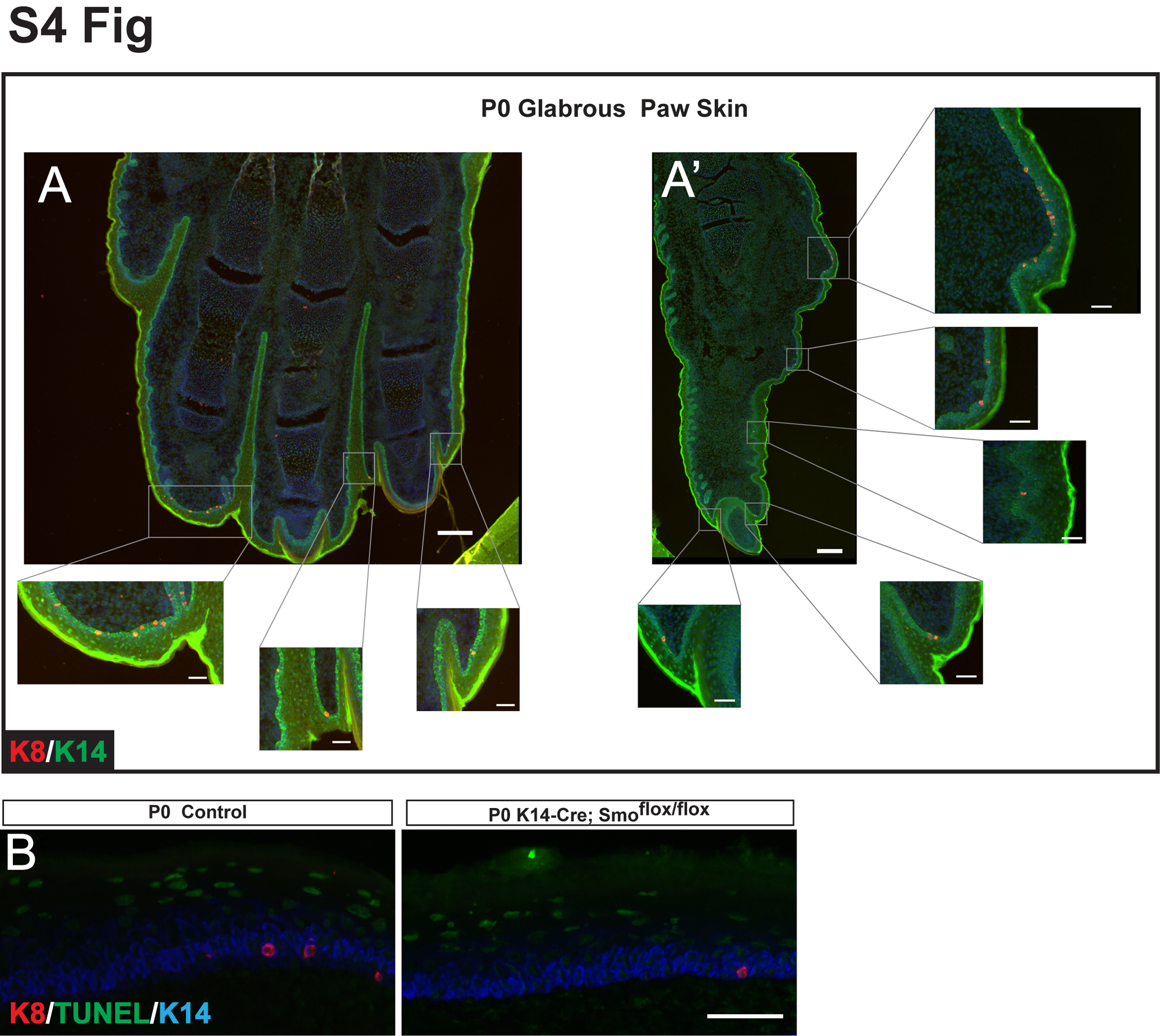

Supplement: S4 Fig — (A) Tile-scan images of paw sections used for Merkel cell quantification in the glabrous paw skin. Krt14(+) (K14) cells were used to quantify the length of the skin in mm, and the number of Krt8(+) (K8) Merkel cells per mm of glabrous paw skin was quantified in magnified images. (B) TUNEL staining showing no defects in apoptosis in the paws of P0 Smo cKO mice compared to control tissue. Note that cells undergoing cornification in the suprabasal layers stain positive for TUNEL as reported previously [33]. Scale bars: (A, A’): 100μm; (A and A’ magnified images, B): 25 μm. (TIF) [file pgen.1006151.s004.tif]

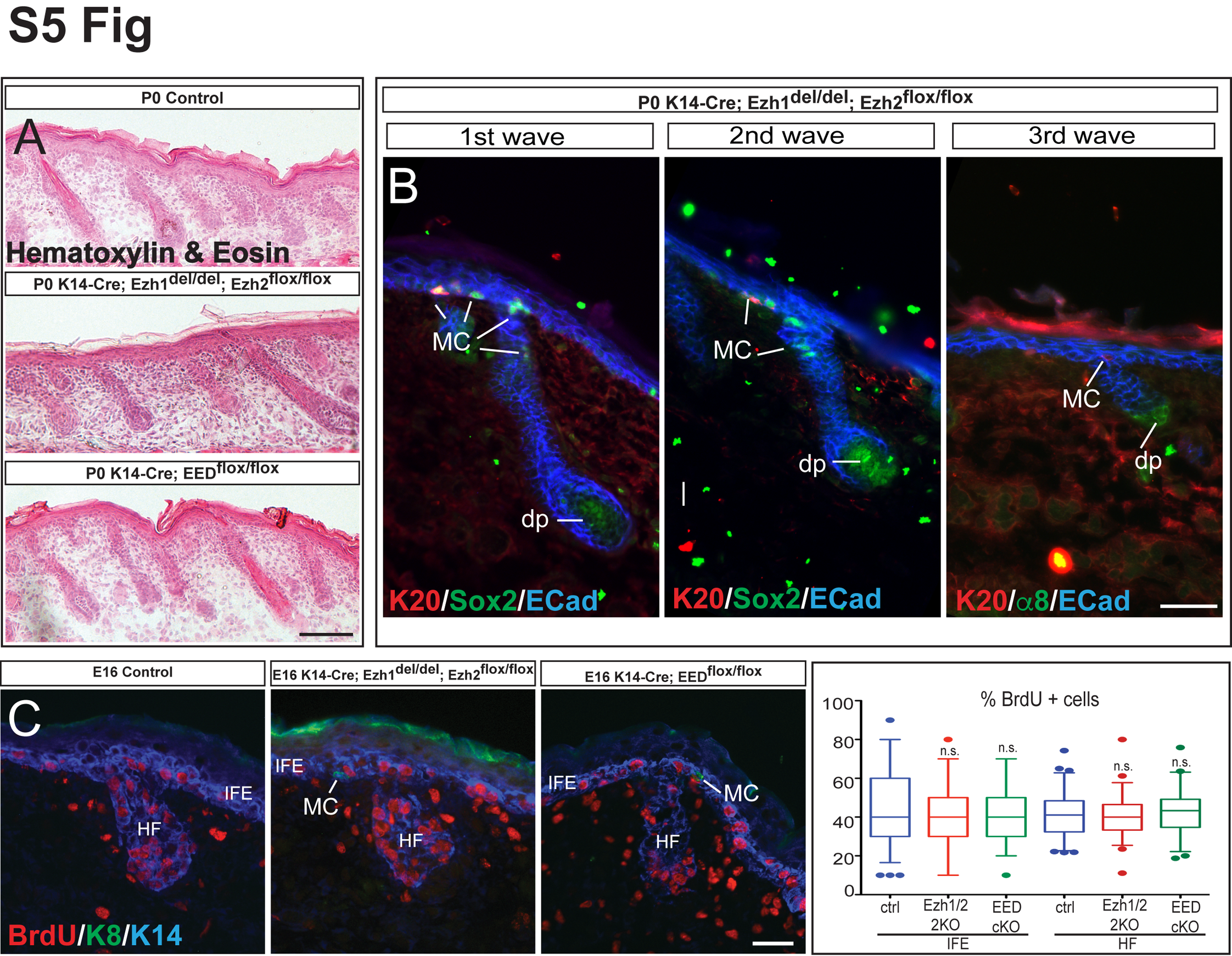

Supplement: S5 Fig — (A) Hematoxylin and Eosin staining showing that hair follicle development is unaffected in the neonatal P0 skin of Ezh1/2 2KO (K14-Cre; Ezh1del/del;Ezh2flox/flox) and EED cKO (K14-Cre; EEDflox/flox) compared to control (ctrl). (B) All hair types can have adjacent Merkel cells (MC) in P0 Ezh1/2 2KO. IF staining for Sox2 and integrin α8 (α8) is used to label the dermal papillae (dp) of different hair follicle types. The dp of first (left) and second (middle) wave hair follicles is Sox2(+), and the two types of hair follicles can be discriminated by size. The dp of the third (right) wave hair follicles is Sox2(-)/α8(+), and these hair follicles are very short at P0. IF staining for Sox2 identifies early-specified Merkel Cells (MC) and Krt20 (K20) identifies mature Merkel cells in the epidermis, which is labeled with E-Cadherin (ECad). (C) IF staining for BrdU shows no significant change in proliferation in the developing interfollicular epidermis (IFE) and hair follicles (HF) in E16 Ezh1/2 2KO and EED cKO compared to control. Quantification of the percentage of BrdU(+) cells in the interfollicular epidermis and hair follicles of E16 control, Ezh1/2 2KO, and EED cKO is presented on the right (IFE, p = 0.9361; HF, p = 0.5707). Scale bars: (A): 100 μm; (B,C): 25 μm. (TIF) [file pgen.1006151.s005.tif]

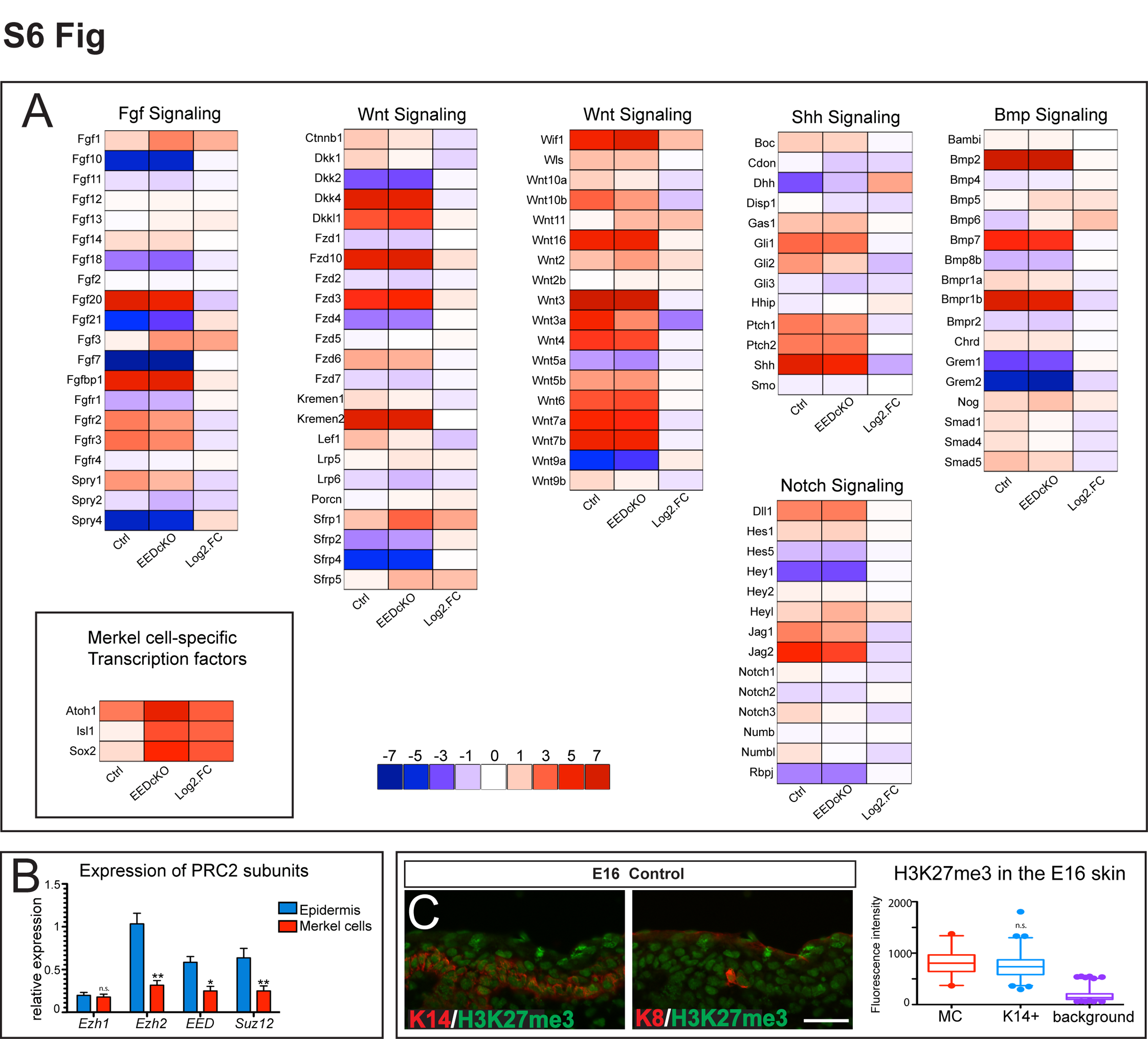

Supplement: S6 Fig — (A) Genes of signaling pathways involved in hair follicle morphogenesis and skin development (Wnt, Shh, FGF, BMP, and Notch signaling) [72], as well as Merkel cell-specific genes were mined from the KEGG database. The expression levels of these genes in P0 control and EED cKO (K14-Cre; EEDflox/flox) skin epithelium (compared to the Universal Mouse Reference RNA) as well as the fold change between the two conditions are represented in heatmaps. (B) RT-qPCR showing significantly less expression of PRC2 subunits Ezh2, EED, and Suz12 in P0 Merkel cells compared to control epidermis (Ezh1, p = 1.0000; Ezh2, p = 0.0011; EED, p = 0.0117; Suz12, p = 0.0041). (C) IF staining showing that Krt8(+) (K8) MCs have the H3K27me3 mark in E16 control Krt14(+) (K14) epidermis. Krt14(+) cells serve as a positive control for H3K27me3 staining. Quantification of H3K27me3 staining intensity (right panel of C) (Kruskal-Wallis test p<0.0001; MC vs K14(+), n.s. p>0.05). Scale bars: (C): 25 μm. (TIF) [file pgen.1006151.s006.tif]

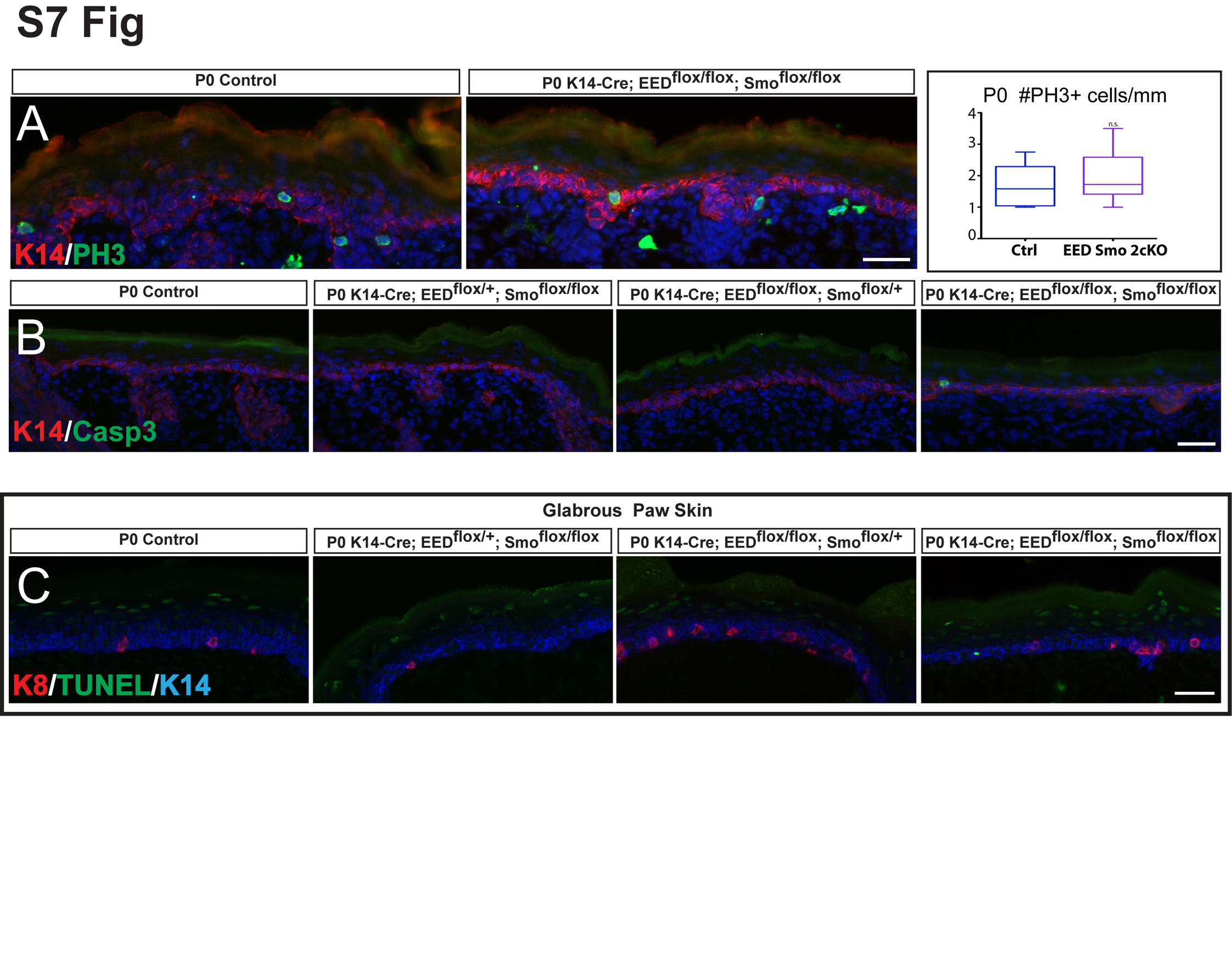

Supplement: S7 Fig — (A) IF staining for the proliferation marker Phospho-Histone H3 (PH3) showing no alterations in proliferation in the skin of P0 EED Smo 2cKO (K14-Cre; EEDflox/flox;Smoflox/flox) mice compared to control (ctrl) epidermis. Quantification of the number of PH3(+) cells (right panel of A) (p = 0.3517). ((B) IF staining for Activated Caspase 3 (Casp3) showing no defects in apoptosis in the dorsal skin of P0 EED Smo 2cKO mice compared to control or EED cKO (K14-Cre; EEDflox/flox;Smoflox/+) mice. (C) TUNEL staining showing no defects in apoptosis in the glabrous skin of P0 EED Smo 2cKO mice compared to control or EED cKO paws. Note that cells undergoing cornification stain positive for TUNEL as reported previously [33]. Unless otherwise indicated, all epidermis represented is dorsal skin. Scale bars: (A-C): 25 μm. (TIF) [file pgen.1006151.s007.tif]
