# Supplementary material for: Polycomb-Mediated Repression and Sonic Hedgehog Signaling Interact to Regulate Merkel Cell Specification during Skin Development
Source: PLoS Genet. 2016 Jul 14;12(7):e1006151. doi: 10.1371/journal.pgen.1006151 (PMC4944976; doi:10.1371/journal.pgen.1006151)
Supplement: S1 Table — (DOCX) [file pgen.1006151.s008.docx]

**S1 Table**

List of real time qPCR primers used for expression and ChIP analyses. All sequences are listed in the 5’ to 3’ direction. Expression primers were used at 0.5 μM for 40 cycles at 60 °C. ChIP primers were used at 0.5 μM for 45 cycles at 60 °C.

**Expression primer sequences**

| **Primer** | **Forward** | **Reverse** |
| --- | --- | --- |
| Sox2 | TTTTGTCCGAGACCGAGAAG | CTCCGGGAAGCGTGTACTTA |
| Atoh1 | GATGGCACAGAAGGACCTGT | TGGCCTCATCAGAGTCACTGTA |
| Isl1 | GAGGGTTTCTCCGGATTTGG | GCGCATTTGATCCCGTACAA |
| PPIB | GTGAGCGCTTCCCAGATGAGA | TGCCGGAGTCGACAATGATG |
| Ezh1 | AGTGGATGCTACCCGGAAAGG | CCCAATGCGGTGATCTCCAT |
| Ezh2 | CAACCCGAAAGGGCAACAAA | AGCAAAGATGCCTATCCTGTGGTC |
| EED | GACCCAAACCTTCTCCTGTCA | TTCATCTCTGTGCCCTTCCA |
| Suz12 | AACTCGAAATCTTATCGCACCAA | TGCAAATGTGCAGACAAGCTAT |
| Gli1 | CTCAGCCCCTCTCCACAGT | CGCTGCTGCAAGAGGACT |
| Gli2 | GACAGCGGGGGCAGAAGTC | TCAGCCTCCCCATGGTTATCTC |
| Gli3 | CCAAGGGGTAACAGGTCCAC | CCACGGGATGCGTACTTACA |
| Shh^+^ | ACGAGGATGGAGCCTGTAGTTTGT | GGGTGTGTGTGGCACGCTTTATTT |
| Patch1 | AAAGAACTGCGGCAAGTTTTTG | CTTCTCCTATCTTCTGACGGGT |
| Wnt3 | GGGCGGCTTCTTTCTCAACTAA | ACAGAATCTCGCCATGGTCTTGT |
| Wnt4 | CTGGAGAAGTGTGGCTGTGA | GGACGTCCACAAAGGACTGT |
| Wnt7a | CCGGGAGATCAAGCAGAAT | TACAGGAGCCTGACACACCA |
| Wnt7b | AGTGCCAGCACCAGTTCC | CCGTGATGGCATAGGTGAAG |
| Wnt10a | GACTCCACAACAACCGTGTG | CCTACTGTGCGGAACTCAGG |
| Wnt10b^+^ | GAACAGCTCTGGGGGTGTAG | GTTCTGGGCTGTAGTGGAGG |
| Fzd10 | GGCTTTGTGGCTTTATTCCA | GAGGGAGAAGACCCCTATGC |
| Tcf3 | CATCCTGGGGCCTTCTCACTTC | GGAGCCGGGGCAACCAGTG |
| Tcf4 | GCCACCTGCGCCCGAGAAT | CACCCGGCCATCGTCACAC |
| Dkkl1^+^ | CCCATGGACTTCCGAGACCTT | CGGGGGCTCTTTTGCTTCTAC |
| Axin2 | AGGAGCAGCTCAGCAAAAAG | CTTCGTACATGGGGAGCACT |
| Sp5 | CGTGAAGACGCACCAAAATA | AGATGTCTTGGGCAGGAGTG |
| Ccnd1 | CCCCAACAACTTCCTCTCCT | TCCAGAAGGGCTTCAATCTG |

+ FROM: Sennett, R., et al. An Integrated Transcriptome Atlas of Embryonic Hair Follicle Progenitors,

Their Niche, and the Developing Skin. Developmental Cell 34, 1-15 (2015).

**ChIP primer sequences**

| **Primer** | **Forward** | **Reverse** |
| --- | --- | --- |
| Sox2 | TGGCCGAATGATTAATAACG | CGTTCTGGCAACAAGTGCTA |
| Isl1 | TTTAAGGCAAAGGTGCACTG | CCTGCTCACGCCTTCATACT |
| Atoh1 | TCAGAATGGAAACAATCATACCAGA | GTTCCCATCGGGATGAGCTA |
| Neuro1 | TCCACTCCCACATGTTCCTT | AACCACCCTTCAAATTCTTCC |
| Olig3 | ACCATCAGGAGAGTCGTCTGAACT | GAAGATCCTGCTCCGACAGCT |
| Chr3 | AACCACCCTTCAAATTCTTCC | GCCCACTGCTATAATTAGGAAGGA |
| Chr5 | CCCTCATCACAGACCCACTTCT | GTGGGAGTGGATGTATCTCTGACTT |
| Actin | GAGGCCGGTGAGTGAGCGAC | TGCGCCGCCGGGTTTTATAGG |
